# Ubiquitin-specific protease 25 ameliorates ulcerative colitis by regulating the degradation of phosphor-STAT3

**DOI:** 10.1038/s41419-024-07315-z

**Published:** 2025-01-07

**Authors:** Zhengru Liu, Jian Liu, Yuping Wei, Jinting Li, Jixiang Zhang, Rong Yu, Qian Yang, Yinglei Miao, Weiguo Dong

**Affiliations:** 1 Department of Gastroenterology, Renmin Hospital of Wuhan University, Wuhan, 430060 China; 2 Department of Gastroenterology, The First Affiliated Hospital of Kunming Medical University, Kunming, 650032 China; 3 Department of Gastroenterology, The Third People’s Hospital of Chengdu, Chengdu, 610000 China; 4 Department of Gastroenterology, Guizhou provincial people’s hospital, Guiyang, 550002 China

**Keywords:** Disease model, Ulcerative colitis

## Abstract

Ubiquitin-specific protease 25 (USP25), a member of the deubiquitination family, plays an important role in protein ubiquitination, degradation, inflammation, and immune regulation. However, the role and mechanism of USP25 in ulcerative colitis (UC) remain unclear. To study the role and mechanism of USP25 in UC, bioinformatics analysis and research are conducted on clinical patients with UC, *Usp25* knockout (*Usp25*^*−/−*^) mice, intestinal epithelial cell-specific knockout signal transducer and activator of transcription 3 (*Stat3)* (Villin-Cre Stat3^fl/fl^) mice, and human colonic epithelial cells. Results show that the expression of USP25 is decreased in patients with UC and mice with dextran sulfate sodium salt (DSS)-induced colitis and that USP25 deficiency exacerbates UC by destroying the intestinal mucosal barrier, however, overexpression of USP25 can alleviate colitis. Mechanistically, USP25 reduces the degradation of phosphor-STAT3^Y705^ at lysine 409 by catalyzing K48-linked deubiquitination. Further, this study demonstrates the aggravation of DSS-induced colitis by intestinal epithelial cell-specific knockout *Stat3* in mice, while *Stat3* overexpression by adeno-associated virus attenuates colitis in DSS-induced *Usp25*^*−/−*^ mice. Together, these results showed that USP25 ameliorates UC by regulating the degradation of phosphor-STAT3. Collectively, USP25 is a specific STAT3 regulator that can be targeted in UC.

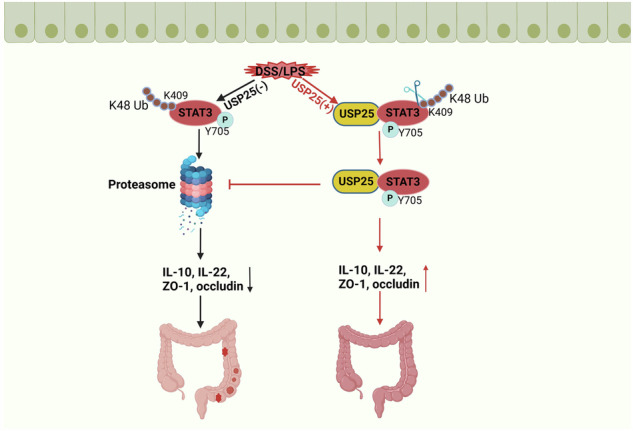

## Introduction

Ulcerative colitis (UC) and Crohn’s disease are two major forms of inflammatory bowel disease (IBD). UC is a chronic inflammatory disease of the colon and rectum [1]. The incidence and prevalence of UC are increasing rapidly worldwide. Over the past few decades, the prevalence of UC has increased in North America, Oceania, and Europe, showing a rapid increase in the newly industrialized countries of Africa, Asia, and South America, which places a heavy economic burden on people and healthcare systems [2]. Although the exact pathogenesis of UC remains unclear, multiple factors have been implicated, including genetic susceptibility, dysregulated immune responses, environmental factors, and altered gut microbiota [3]. Genetic factors are closely associated with UC [4, 5], therefore, the underlying mechanisms of genes in UC progression need to be explored to identify potential molecular targets for treatment.

Post-translation modification of protein includes ubiquitination, depubiquitination, phosphorylation, acetylation, palmitoylation and so on. Ubiquitin is a reversible post-translational modification, which is regulated by E3 Ubiquitin Ligase and Deubiquitin Enzyme. As one of the most important post-translational modifications, ubiquitination is involved in the pathogenesis of various intestinal diseases, such as inflammatory bowel disease [6]. In the process of UC, deubiquitination enzyme removes the ubiquitin chains covalently bound to the substrate protein, which regulates the degradation, activation and localization of the substrate protein, which also regulates the interaction between proteins. After being modified by ubiquitination, protein can be protected from being degraded by ubiquitin proteasome, which increasing its stability [7]. Phosphorylation modification is the final form of STAT3 activation, which mainly includes tyrosine and serine phosphorylation sites.STAT3 phosphorylation is involved in the occurrence and development of various colon diseases and also plays an important role in UC [8–10].

Deubiquitinase (DUB)-mediated deubiquitination processes are closely associated with the occurrence and development of colonic inflammation.Previous study showed that the deubiquitinase OTUD1 inhibits colonic inflammation by suppressing RIPK1-mediated NF-κB signaling [11]. Besides, another study showed that mice with cell-specific USP8 deficiency developed colitis that was promoted by disturbed T cell homeostasis [12]. Ubiquitin-specific protease 25 (USP25) is a deubiquitinase that removes the poly-ubiquitin chain from selected substrate proteins to spare them from proteasome recognition and degradation [13]. USP25 hydrolyzes K48 and K63 ubiquitin chains linked to substrate proteins [14]. USP25 promotes HBO1 stability during bacterial infection, thereby enhancing the HBO1-mediated transcription of inflammatory genes [15]. Additionally, USP25 deubiquitinates and stabilizes hypoxia-inducible factor (HIF)-1α transcription factor, which promotes pathological HIF-1-driven metabolic reprogramming. Moreover, USP25 is a potential therapeutic target in pancreatic cancer [16]. and plays an important role in Alzheimer’s disease by regulating amyloid precursor protein processing and Aβ production [17]. Genome sequencing studies show that the expression level of USP25 in the colonic mucosa of patients with UC is lower than that in healthy individuals [4, 5]. Nevertheless, the pathogenesis of USP25 in UC requires further investigation.

Signal transducer and activator of transcription 3 (STAT3) plays an important role in regulating inflammation and the immune response. STAT3 is an essential cytoplasmic transcription factor for the downstream signal transduction pathway of gp130, which includes interleukin (IL)-6 family cytokine receptors and IL-10/IL-22 receptors [8–10]. STAT3 deficiency in mice induces colitis [18]. Phosphorylation activation of STAT3 mediates the production of the anti-inflammatory factor IL-10/IL-22, thereby suppressing intestinal inflammation in inflammatory bowel disease [19]. In addition, STAT3 phosphorylation promotes intestinal epithelial regeneration [20, 21]. The deubiquitinating enzyme USP28 mediates STAT3 signaling in non-small-cell lung cancer cells, and USP28 interacts with STAT3 and enhances its stability by inducing the deubiquitination of STAT3 [22]. Study by Shrma et al. showed that STAT3 phosphorylation can up-regulate the expression of tight junction protein in colitis mice, which promote intestinal barrier integrity and prevent colon inflammation and tumorigenesis [23]. And the intestinal mucosal barrier plays a vital role in ulcerative colitis. However, currently, no studies on the regulation of STAT3 expression by USP25 in colitis exist.

In this study, we showed that USP25 was downregulated in patients with UC and dextran sulfate sodium salt (DSS)-induced colitis. Mechanistically, USP25 interacts with phosphorylated STAT3 (p-STAT3), and USP25 reduces the degradation of phosphor-STAT3^Y705^ at lysine 409 by catalyzing K48-linked deubiquitination. These results suggest that USP25 is a specific STAT3 regulator that can be targeted in UC.

## Methods

### Bioinformatics analysis

GSE47908 (UC = 45, control = 15), GSE38713 (UC = 30, control = 13), and GSE9452 (UC = 21, control = 5) datasets were downloaded from the GEO database. These three datasets include gene expression data from the colonic mucosa of healthy controls and patients with UC. All patients with UC had their diagnosis verified by established criteria [24], and they were graded in accordance with the Mayo score when biopsies were obtained.

The endoscopic assessment of activity was confirmed by a histological examination, and any discrepancy between the observations led to exclusion of the specific patient. Exclusion criteria were as followings: age below 18 or above 80 years, recent (within 14 d) intake of antibiotics or probiotics, clinical evidence of infection, mental illness or pregnancy.

We extracted and compared the colonic USP25 expression of healthy controls and patients with UC from these datasets.

### Human colonic tissue collection

Colonic tissues from patients with UC and healthy controls were collected from the Department of Gastroenterology at the Renmin Hospital of Wuhan University. Informed consent was obtained from all patients and healthy controls. This study was approved by the Ethics Committee of Renmin Hospital of Wuhan University (No. 2018 K-C089).

### Colitis induction in mice

The animal experimental protocol was approved by the Animal Ethics Committee of Renmin Hospital of Wuhan University (No. WDRY 2018-K033). C57BL/6 WT mice were purchased from Vital River Laboratory (Beijing, China). C57BL/6 *Usp25* knockout mice (*Usp25*^*−/−*^) were provided by Professor Dong Cheng (Institute for Immunology and School of Medicine, Tsinghua University, Beijing, China). IEC-specific knockout *Stat3* (Villin-Cre *Stat3*^fl/fl^) and *Stat3*^fl/fl^ mice were purchased from the Model Animal Research Center of Nanjing University (Nanjing, China). All experimental animals were housed in a specific pathogen-free animal facility at the Animal Experiment Center at the Renmin Hospital of Wuhan University.

Agarose gel electrophoresis (2%) was used to determine the genotypes of experimental mice. The male mice (six to eight weeks of age, 20 ± 0.8 g, *n* = 8 per group) were randomly divided into different groups. The experimental mice continuously drank distilled water containing 3% DSS (MP Biomedicals, Irvine, CA, USA) for seven days. The body weight of the mice and stool characteristics were recorded, and the presence of blood in the stool of the mice was recorded daily. Blood was collected after the mice were anesthetized with isoflurane. Experimental mice were sacrificed, colons were removed immediately, and the length of the colons was recorded. The mouse disease activity index (DAI) was based on mouse stool hardness and fecal occult blood tests. Scores were allocated as described in Table 1. The DAI was determined by summing the scores for each category.

**Table 1:** DAI scoring standard of experimental mice.

Stool hardness	Fecal occult blood tests	Score
Normal stool	No color change after 3 min	0
Slightly soft stool	Light blue in 1–3 min	1
Soft stool	gradually become blue in 0–60 s	2
Loose stool	immediately appear blue	3
Watery stool	Immediately appear dark blue	4

The mouse disease activity index (DAI) was based on mouse stool hardness and fecal occult blood tests. Scores were allocated as described in Table 1. The DAI was determined by summing the scores for each category.

### Histological examination

The colonic tissue was stained with hematoxylin and eosin. Histological scores were calculated by three pathologists. Histological scores were determined blindly as described in Table 2. The total histopathological score was determined by summing the scores for each category.

**Table 2:** Colon histological score of experimental mice.

Score	Damaged area	Tissue damage	Muco-depletion of glands	Inflammatory cell infiltration
0	n/a	No mucosal damage	None	Occasional inflammatory cells in the lamina propria
1	≤25%	Discrete epithelial lesions	Mild	Increased numbers of inflammatory cells in the lamina propria
2	≤50%	Surface mucosal erosion or focal ulceration	Moderate	Confluent inflammatory cells extending into the submucosa
3	≤75%	Extensive mucosal damage and extension into deeper structures of the bowel wall	Moderate	Transmural extension of the infiltrate
4	≤100%	–	Severe	–

The colonic tissue was stained with hematoxylin and eosin. Histological scores were calculated by three pathologists. Histological scores were determined blindly as described in Table 2. The total histopathological score was determined by summing the scores for each category.

### Immunochemistry

After deparaffinization, hydration, antigen retrieval, and serum blocking, colon sections were incubated with the following primary antibodies overnight at 4 °C: USP25 (ABclonal Technology, Woburn, MA, USA) and STAT3 (Cell Signaling Technology, Danvers, MA, USA). Goat anti-rabbit horseradish peroxidase (Aspen Pharmacare, Durban, South Africa) was added to the colon slices.

### Immunofluorescence

After deparaffinization and serum blocking, colon sections were incubated with ZO-1 antibody (Proteintech, Rosemont, IL, USA) at 4 °C. NCM460 cells were incubated with ZO-1 (Proteintech), occludin (Cell Signaling Technology) and p-STAT3^Y705^ (Cell Signaling Technology) antibodies at 4 °C. After washing with PBS, CY3-labeled goat anti-rabbit antibody (Aspen Pharmacare) was added to colon sections and cells, and 4,6-diamino-2-phenylindole (DAPI, Aspen Pharmacare) was used to stain the nuclei. Image J (National Institutes of Health, Bethesda, MD, USA) was used to measure the fluorescence intensity of ZO-1, occludin and p-STAT3.

### Western blotting

A total protein extraction kit (Beyotime Biotechnology, Shanghai, China) and BCA kit (Beyotime Biotechnology) were used to extract total protein and determine the protein concentration. After electrophoresis, proteins were transferred to polyvinylidene fluoride membranes and blocked, following which, membranes were incubated for 12 h at 4 °C with the following antibodies: glyceraldehyde 3-phosphate dehydrogenase (GAPDH) (Cell Signaling Technology, #5174), USP25 (ABclonal Technologies, #A23431), ZO-1 (Proteintech, #21773-1-AP), occludin (Proteintech, #13409-1-AP). STAT3 (Cell Signaling Technology, #9139), and p-STAT3 (Tyr705) (Cell Signaling Technology, #9145), Ubiquitin (Cell Signaling Technology, #20326), HA (Cell Signaling Technology, #3724), Flag (Cell Signaling Technology, #14793), Myc (Cell Signaling Technology, #2278). Membranes were washed and incubated with a secondary mouse/rabbit antibody (Proteintech, #SA00001-1 or #SA00001-2).

### Enzyme-linked immunosorbent assay

Mouse blood samples were centrifuged at 3000 × *g* at 4 °C for 10 min, and before examination, the serum was stored at −80 °C. Serum IL-10 and IL-22 levels were determined using mouse IL-10 and IL-22 enzyme-linked immunosorbent assay (ELISA) kits (Quantikine; R&D Systems, Minneapolis, MN, USA) according to the manufacturer’s instructions. All serum samples were analyzed in triplicate.

### Cell culture

The human normal colonic epithelial cell line NCM460, Caco2, and human embryonic kidney 293T cell lines were purchased from the American Type Culture Collection (Manassas, VA, USA). All cells were cultured in Dulbecco’s modified Eagle’s medium (DMEM; Sigma-Aldrich, St. Louis, MO, USA) supplemented with 10% fetal bovine serum (FBS; Gibco, Waltham, MA, USA) and 1% penicillin and streptomycin (Invitrogen, Waltham, MA, USA) at 37 °C and 5% CO_2_.

### Plasmid construction and viral infection

All lentiviruses or adenoviruses targeting USP25, STAT3, and WT or mutant plasmids were purchased from GenePharma Genomics (Suzhou, China). Cell transfection was performed according to the manufacturer’s instructions. Tail-vein injection of an adeno-associated virus was used to induce *Usp25* overexpression in mice. Cells were cultured using Lipofectamine 2000 (GenePharma Genomics) and transfected with plasmids according to the manufacturer’s instructions.

### Immunoprecipitation

Immunoprecipitation of proteins was performed using the EpiZyme Protein A/G Immunoprecipitation Kit (Shanghai, China), according to the manufacturer’s instructions. Cell proteins were extracted using IP lysis buffer after transfecting with the indicated plasmids in 293T cells. For endogenous IP, cells were lysed using IP lysis buffer. Samples were incubated with protein A/G agarose beads and the indicated primary antibodies. After washing the agarose beads with IP lysis buffer and centrifugation, the loading buffer was added to the protein and boiled for 10 min. Finally, western blotting was performed as previously described.

### Mass spectrometry analysis

USP25 and its interacting proteins were immunoprecipitated using the IP assay described above. Immunoprecipitated proteins were subsequently analyzed using liquid chromatography–tandem mass spectrometry (LC-MS/MS). The criterion for selecting the candidate molecules was that the number of unique peptides should be >2.

#### Statistical analysis

GraphPad Prism software version 8.0 was used to perform statistical analyses. An unpaired Student’s *t* test was used to compare normally distributed parameters between the two groups. Wilcoxon rank–sum tests were used to compare non-normally distributed parameters. For multi-group testing, we used one- or two-way analysis of variance (ANOVA) combined with Bonferroni’s posthoc test. Statistical significance was defined as *p* < 0.05.

#### Ethics approval statement

Informed consent was obtained from all patients and healthy controls. This study was approved by the Ethics Committee of Renmin Hospital of Wuhan University (No. 2018 K-C089).The animal experimental protocol was approved by the Animal Ethics Committee of Renmin Hospital of Wuhan University (No. WDRY 2018-K033).

## Results

### Decreased USP25 expression in ulcerative colitis

**Fig. 1:**
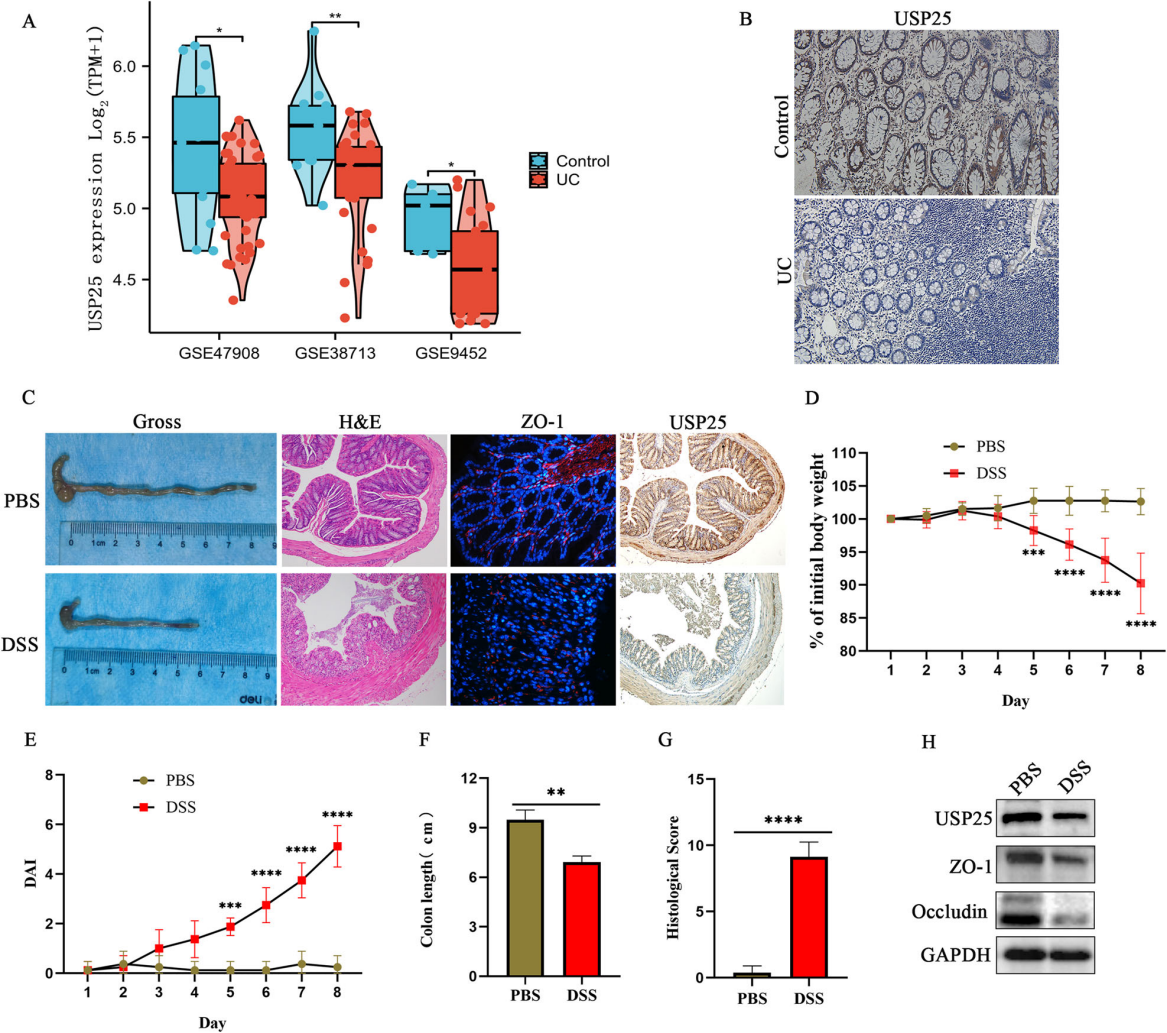
The expression of USP25 is decreased in ulcerative colitis. **A** USP25 expression in patients with UC and healthy controls from the GSE47908, GSE38713, and GSE9452 datasets from the GEO database were compared and analyzed. **B** Immunohistochemistry for USP25 was performed on human colonic tissue from patients with UC (*n* = 8) and healthy controls (*n* = 8). **C** Mice were treated with DSS for seven days (*n* = 8 per group). Mouse colons were photographed and recorded and stained with hematoxylin and eosin (*n* = 8 per group), ZO-1 immunofluorescence staining (*n* = 5 per group), and USP25 immunohistochemical staining (*n* = 5 per group). Induction of colitis in mice was assessed by changes in mouse body weight (**D**), DAI score (**E**), colon length (**F**), and histopathological score (**G**). Body weight changes in mice are expressed as a percentage of the initial body weight (**D**). **H** Western blotting was performed to examine the protein expression levels of USP25, ZO-1 and occludin in the colons of control and UC mice. **p < 0.01, ***p < 0.001 compared with control groups.

To explore the expression of USP25 in human UC intestinal mucosa, we downloaded the GSE47908, GSE38713, and GSE9452 datasets from the GEO database. As shown in Fig. 1A, colonic USP25 expression in patients with UC was significantly lower than that in healthy controls (*p* < 0.05). Consistent with this, immunohistochemical staining of USP25 in human colonic mucosa showed that the expression of USP25 in the colonic mucosa of patients with UC was significantly reduced compared with that in healthy controls (*p* < 0.05) (Fig. 1B, Supplementary Fig. S1A).

To further investigate the expression of USP25 in UC animal models of UC, DSS was used to successfully induce colitis in mice. As shown in Fig. 1C–G, in the UC mice, the colons were significantly shortened (*p* < 0.05), body weight was significantly decreased (*p* < 0.05), disease activity index (DAI) (*p* < 0.05) and colon histopathological scores (*p* < 0.05) were significantly increased, and inflammatory damage, tissue damage, muco-depletion of glands, and infiltration of inflammatory cells were observed in the colonic tissue. In addition, the expression of the tight junction protein ZO-1 and occludin in the colonic tissue of mice in the UC group were significantly lower than those in the control group (*p* < 0.05) (Fig. 1C, H and Supplementary Fig. S1B). Moreover, the expression of USP25 in the colonic tissue of mice in the UC group was significantly lower than that of those in the control group (*p* < 0.05) (Fig. 1C, H, and Supplementary Fig. S1C). In summary, the expression of USP25 is downregulated in UC.

### USP25 deficiency exacerbates ulcerative colitis in vitro and in vivo

**Fig. 2:**
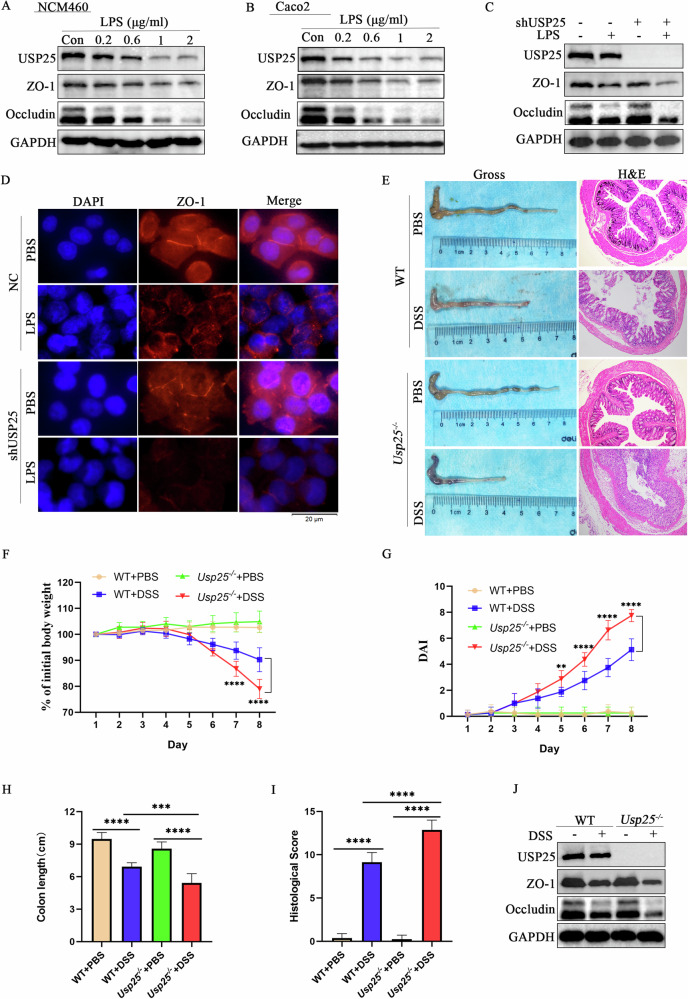
USP25 deficiency exacerbates ulcerative colitis in vitro and in vivo. **A**, **B** To determine the optimal LPS concentration, LPS was used to induce cellular inflammation in the human colonic epithelial cell lines NCM460 and Caco2 cells. **C** After using lentivirus shUSP25 to interfere with the expression of USP25 in NCM460 cells, the cells were co-cultured with LPS. Western blotting was performed to examine the protein expression level of USP25 and ZO-1. **D** Immunofluorescence was performed to examine the expression of ZO-1 protein in control and USP25 low-expressing NCM460 cells with or without LPS treatment. **E** DSS was used to induce colitis in wild-type and *Usp25*^*−/−*^ mice (*n* = 8 per group). Mice colons were photographed and recorded, and colons were stained with hematoxylin and eosin (*n* = 8 per group). Induction of colitis in mice was assessed by changes in mice body weight (**F**), DAI score (**G**), colon length (**H**), and histopathological score (**I**). Body weight change in mice was expressed as a percentage of initial body weight (**F**). **J** Western blotting was performed to examine the protein expression level of USP25, ZO-1 and occludin in mice colons of four groups. **p < 0.01, ***p < 0.001 and ****p < 0.0001 compared with control groups.

To further investigate the function of USP25 in UC, lipopolysaccharide (LPS) was used to induce cellular inflammation in the human colonic epithelial cell lines NCM460 and Caco2 (Fig. 2A, B). The results showed that when the concentration of LPS was 1 μg mL^−1^, the expression of the tight junction protein ZO-1 and occludin in cells were significantly decreased (*p* < 0.05), and the expression of USP25 was significantly downregulated (*p* < 0.05) (Fig. 2A, B). In addition, after using lentivirus shUSP25 to interfere with the expression of USP25 in NCM460, cells were co-cultured with LPS (1 μg mL^−1^), and the expression of the tight junction protein ZO-1 in cells with low USP25 expression were significantly lower than that in the control group (*p* < 0.05) (Fig. 2C, D and Supplementary Fig. S1D). Therefore, decreased USP25 expression in cells aggravates tight junction damage and inflammatory damage.

To further study the contribution of USP25 to the pathogenesis of UC in vivo, we examined DSS-induced UC in *Usp25*^*−/−*^ mice. The identification of *Usp25* genotypes is shown in Supplementary Fig. S1E. Compared with wild-type (WT) mice, *Usp25*-deficient UC-group mice showed significantly shorter colon length (*p* < 0.05), significant weight loss (*p* < 0.05), and increased DAI (*p* < 0.05) and histopathological scores (*p* < 0.05) (Fig. 2E–I). Moreover, serum levels of the anti-inflammatory factors IL-10 and IL-22 in *Usp25*-deficient UC mice were lower than those in the WT UC mice (Supplementary Fig. S1F, G). In addition, *Usp25*-deficient UC group mice also exhibited more severe tight junction damage than WT UC mice (*p* < 0.05) (Fig. 2J). Thus, a USP25 deficiency aggravates UC in vitro and in vivo.

### USP25 overexpression alleviates ulcerative colitis in vitro and in vivo

**Fig. 3:**
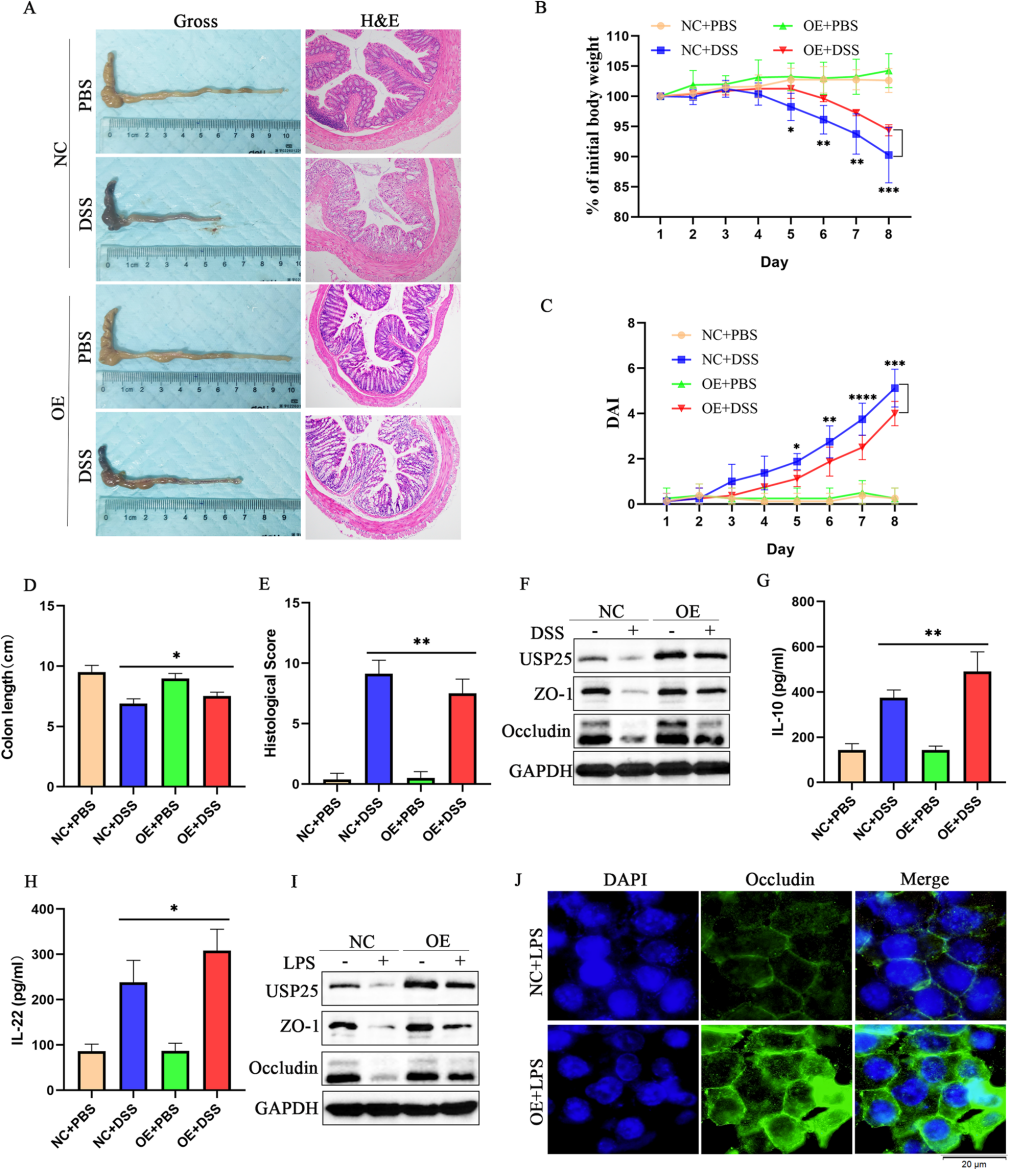
USP25 overexpression alleviates ulcerative colitis in vitro and in vivo. **A** Adeno-associated virus 8 (AAV8) expressing Usp25 (AAV–Usp25) was injected into mice to overexpress USP25 and subsequently induce colitis in mice with DSS. Colons were photographed and recorded and stained with hematoxylin and eosin (*n* = 8 per group). Induction of colitis in mice was assessed by changes in mouse body weight (**B**), DAI score (**C**), colon length (**D**), and histopathological score (**E**). Body weight change in mice was expressed as a percentage of the initial body weight (**B**). **F** Western blotting was performed to examine the protein expression levels of USP25, ZO-1 and occludin in colons from the mice in four groups. **G**, **H** The levels of mouse serum inflammatory factors (IF) IL-10 and IL-22 were measured by enzyme-linked immunosorbent assay. **I** After using the plasmid to overexpress USP25 in NCM460 cells, the cells were co-cultured with LPS. Western blotting was performed to examine the protein expression levels of USP25, ZO-1 and occludin. **J** Immunofluorescence was performed to examine the expression of occludin protein in NC and USP25 overexpressing NCM460 cells with LPS treatment. *p < 0.05, **p < 0.01 compared with control groups.

To further verify the protective effect of USP25 in colitis, we examined the therapeutic effect of *Usp25* in DSS-induced colitis in mice. An adeno-associated virus 8 (AAV8) expression vector *Usp25* (AAV**–***Usp25*) was used to overexpress USP25 and subsequently induce colitis in DSS mice. The results are shown in Fig. 3A–F. Compared with the control mice, UC mice overexpressing USP25 exhibited longer colons (*p* < 0.05), less weight loss (*p* < 0.05), lower DAI scores (*p* < 0.05) and histopathological scores (p < 0.05), and less tight junction disruption (*p* < 0.05). Moreover, serum levels of anti-inflammatory factors IL-10 and IL-22 in the USP25 overexpression UC mice were higher than those in the NC–UC mice (Fig. 3G–H). Similarly, in LPS-induced NCM460 cells, overexpression of USP25 increased the expression of tight junction ZO-1 and occludin (Fig. 3I, J). Therefore, USP25 alleviates UC by protecting tight junctions in vitro and in vivo.

### USP25 interacts with STAT3

**Fig. 4:**
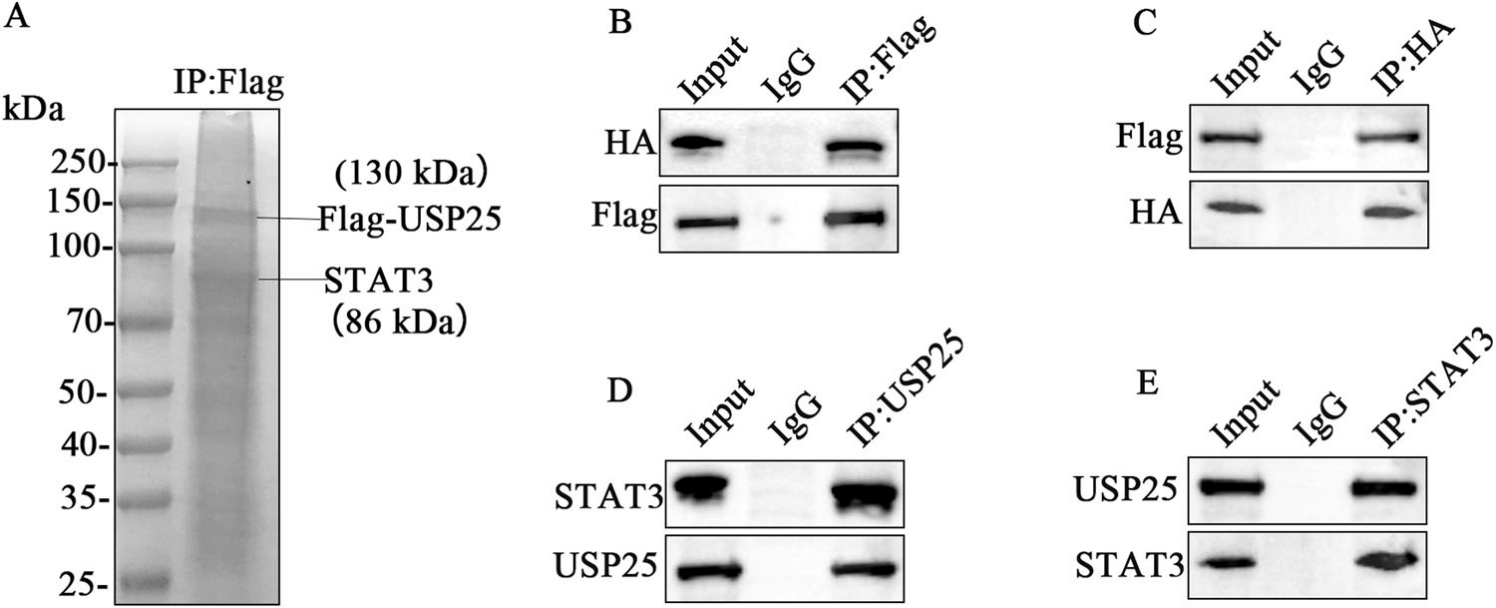
USP25 interacts with STAT3. **A** IP–MS analysis was performed to identify the specific target mediating the suppression of inflammation by USP25 in USP25-overexpressing 293T cells. **B**, **C** The overexpression plasmids Flag-USP25 and HA-STAT3 were transfected into 293T cells. The protein interaction between USP25 and STAT3 was verified by co-immunoprecipitation (IP-Flag and IP-HA, respectively). **D**, **E** The interaction between endogenous USP25 and endogenous STAT3 was verified in the human intestinal epithelial cell line NCM460.

To identify the specific target mediating the suppression of inflammation by USP25, we performed immunoprecipitation–mass spectrometry (IP**–**MS) analysis using USP25-overexpressing 293T cells (Fig. 4A and Supplementary Table 1). Multiple proteins associated with the regulation of inflammation were detected in the results of the mass spectrometry analysis, including STAT3, HSP90A, and BIP. Previous studies showed that STAT3 plays an important role in the pathogenesis of UC [14], and STAT3 is regulated by the deubiquitinase USP28 [18]. However, the regulatory role of USP25 in STAT3 in UC is still unknown. Accordingly, STAT3 was selected as a candidate USP25 target. To confirm the protein interaction of USP25 with STAT3, as shown in Fig. 4B, C, we transfected the overexpression plasmids Flag-USP25 and HA-STAT3 in 293T cells and verified the protein interaction between USP25 and STAT3 by co-immunoprecipitation (co-IP) (IP-Flag and IP-HA, respectively). This result was further validated in human intestinal epithelial cells NCM460 (IP-USP25 and IP-STAT3, respectively) (Fig. 4D, E). USP25 interacts with STAT3; however, the specific mechanism requires further study.

### USP25 promotes K48-linked deubiquitination of STAT3

**Fig. 5:**
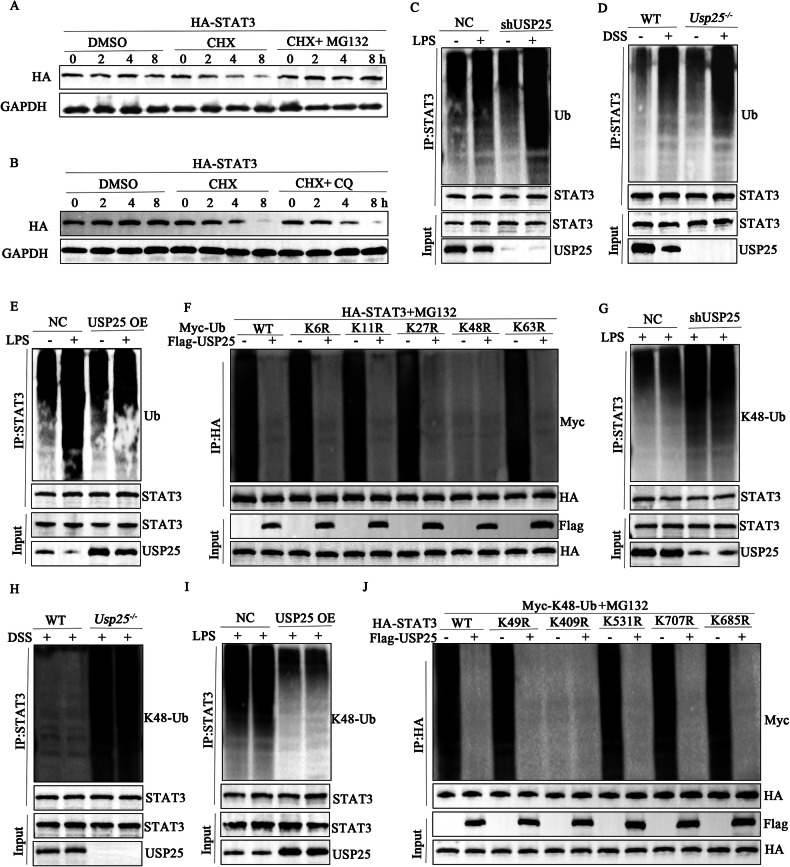
USP25 promotes K48-linked deubiquitination of STAT3. **A**, **B** Plasmids overexpressing HA-STAT3 were transfected into 293T cells. Cycloheximide was used to inhibit protein synthesis, and MG132 and chloroquine were used to inhibit ubiquitin-lysosome and autophagolysosomes, respectively, to degrade proteins. The time gradient was set to 0, 2, 4, and 8 h. Western blotting was performed to examine STAT3 protein levels corresponding to each time point. Protein ubiquitination assays were performed on NCM460 cells with low USP25 expression (**C**) or USP25-overexpressing NCM460 cells (**E**) with or without LPS treatment and the intestinal tissue of WT/*Usp25*^*−/−*^mice with or without DSS treatment (**D**). **F** In screening for potential lysine ubiquitination types, the deubiquitination of HA-STAT3 in response to USP25 overexpression was examined in 293T cells transfected with the wild-type and mutated Myc-Ub plasmids. **G**–**I** Ubiquitination assays determining the deubiquitination of endogenous STAT3 in NCM460 cells with low USP25 expression (**G**) or USP25-overexpressing NCM460 cells (**H**) with LPS treatment and colons of WT/ *Usp25*^*−/−*^mice with DSS administration. **J** Ubiquitination assays determining the deubiquitination of HA-STAT3-K49R/K409R/K531R/K707R/K685R in response to USP25 overexpression in 293T cells transfected with the indicated plasmids.

To further explore the degradation of STAT3 and the regulation of STAT3 by USP25 in UC, we overexpressed HA-STAT3 in 293T cells. Cycloheximide (CHX) was used to inhibit protein synthesis. MG132 and chloroquine (CQ) were used to inhibit ubiquitin-lysosome and autophagolysosomes, respectively, to degrade proteins, and we set the time gradient to 0, 2, 4, and 8 h and examined STAT3 levels at each time point. The results showed that after cells were cultured with the MG132 inhibitor, levels of STAT3 protein did not change with time; however, CQ could not prevent the degradation of STAT3, suggesting that the degradation of STAT3 protein is dependent on the ubiquitin**–**proteasome pathway (Fig. 5A, B).

Since USP25 acts as a deubiquitinase, which can inhibit protein degradation by deubiquitination, we speculate that USP25 may affect the ubiquitinated degradation of STAT3. To verify the results of ubiquitinomics, we performed protein ubiquitination assays and found that USP25 facilitated the deubiquitination of STAT3 in NCM460 cells and mouse intestinal tissue. As shown in Fig. 5C, in NCM460 cells with low USP25 expression, STAT3 was degraded more by ubiquitination, and this result was further verified in the colonic tissue of *Usp25*^*−/−*^ mice (Fig. 5D). In contrast, ubiquitinated degradation of STAT3 was hampered in USP25-overexpressing NCM460 cells (Fig. 5E).

Next, we screened mutated forms of deubiquitin for potential lysine ubiquitination types in 293T cells. USP25 failed to link K48R (only Lys48 was mutated) to STAT3 (Fig. 5F), indicating that USP25 predominantly promoted K48-linked deubiquitination of STAT3. We observed that K48-linked ubiquitination was increased in USP25-downregulated NCM460 cells and the colonic tissue of *Usp25*^*−/−*^ mice (Fig. 5G, H) but decreased in USP25-overexpressing NCM460 cells (Fig. 5I). To determine the potential USP25-catalyzed lysine sites, the five lysine sites of STAT3 were mutated separately according to the predicted results of ubiquitinomics (STAT3-K49R/K409R/K531R/K707R/K685R). Results showed that USP25 promoted K48-linked deubiquitination of STAT3- K49R /K531R/K707R/K685R in 293T cells, whereas the mutation of K409 abolished K48-linked deubiquitination and USP25-mediated the deubiquitination of STAT3, indicating that the K409 site was the specific site responsible for USP25-catalyzed deubiquitination of STAT3 (Fig. 5J).

### USP25 attenuates the proteasomal degradation of phosphor-STAT3

**Fig. 6:**
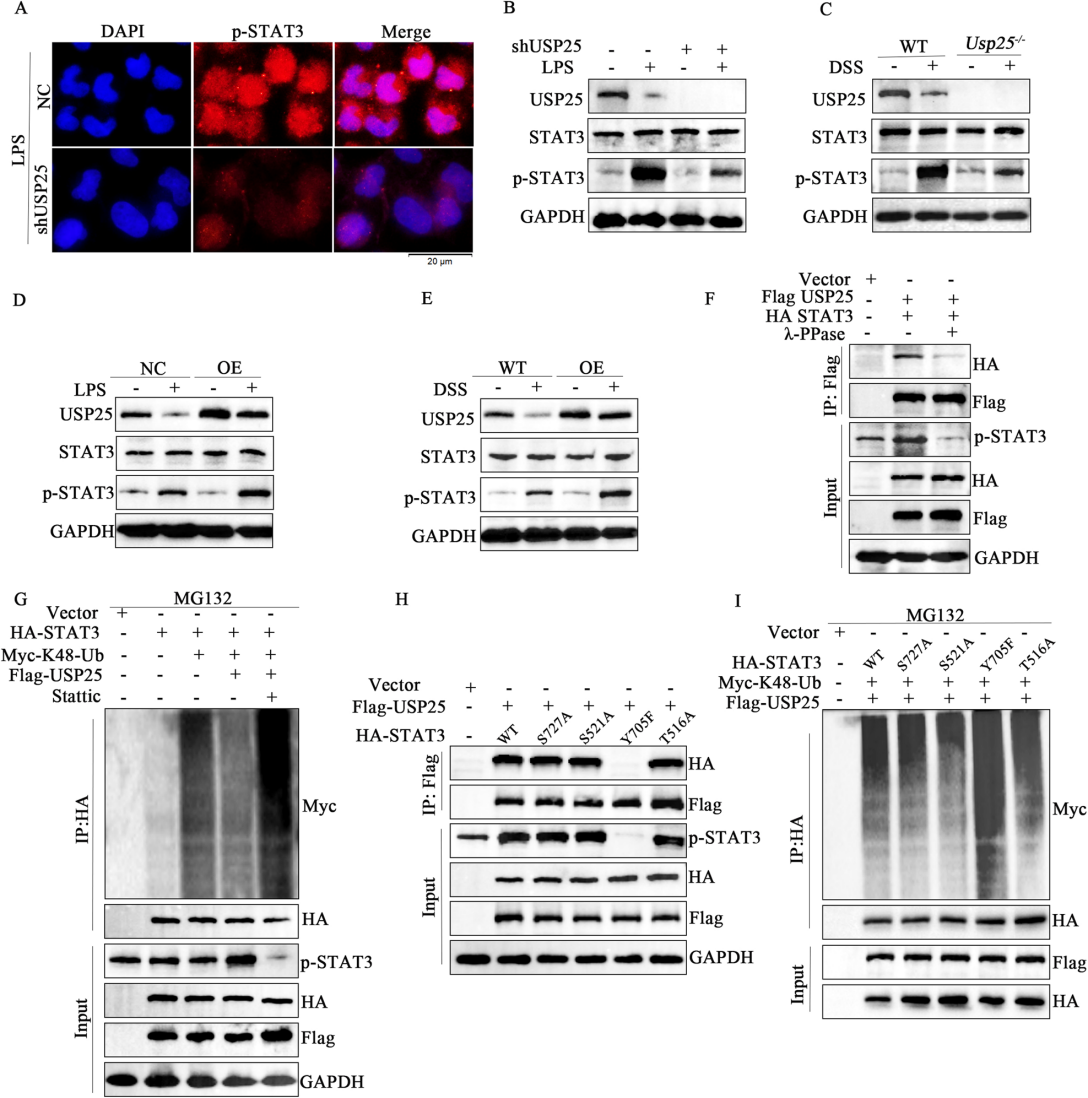
USP25 attenuates the proteasomal degradation of phospho-STAT3. **A** Immunofluorescence was performed to examine the expression of p-STAT3 in NCM460 cells with or without USP25 low expression. Western blotting was performed to examine the indicated proteins in control groups vs. USP25 low-expressing NCM460 cells (**B**), colon tissues of *Usp25*^*−/−*^ mice (**C**), USP25 overexpressing NCM460 cells (**D**), and USP25 overexpressing mice (**E**), with or without LPS/DSS treatment. **F** Co-immunoprecipitation assays showing the interaction between STAT3 and USP25 in 293T cells transfected with the indicated plasmids, with or without lambda protein phosphatase treatment. **G** Protein ubiquitination assays were performed in 293T cells transfected with the indicated plasmids, with or without static treatment. **H** Co-immunoprecipitation assays showing the interaction of USP25 with HA-STAT3-WT and mutation HA-STAT3-S727A/S521A/Y705F/T516A in 293T cells transfected with the indicated plasmids. **I** Ubiquitination assays to determine the deubiquitination of WT and mutated STAT3-S727A/S521A/Y705F/T516A in response to USP25 overexpression in 293T cells transfected with the indicated plasmids.

Next, we investigated the impact of USP25-catalyzed STAT3 deubiquitination. Immunofluorescence analysis showed that the expression of p-STAT3 was reduced in USP25-downregulated NCM460 cells (Fig. 6A). Western blot results also confirmed that the expression of phosphorylated STAT3 was reduced in USP25-downregulated NCM460 cells and colonic tissues of *Usp25*^*−/−*^ mice, whereas the expression of phosphorylated STAT3 was increased in USP25-overexpressing NCM460 cells; however, no significant difference in the expression of total STAT3 was observed (Fig. 6B–E). These results suggest that USP25 promotes the deubiquitination of phosphorylated STAT3 instead of total STAT3. As expected, when lambda protein phosphatase (λ-PPase) was utilized to inhibit protein phosphorylation, the interaction between USP25 and STAT3 was significantly reduced (Fig. 6F). Furthermore, after stattic, a phosphorylation inhibitor of STAT3, was utilized to inhibit STAT3 phosphorylation, the expression of p-STAT3 was reduced, and USP25-catalyzed K48-linked STAT3 deubiquitination was attenuated. Thus, ubiquitination and degradation of STAT3 were increased (Fig. 6G).

These findings suggest that USP25 physically interacts with p-STAT3. To further confirm this possibility and identify potential phosphorylation sites of STAT3, according to the predicted results of phosphorylation sites, four phosphorylation sites (S727, S521, Y705, and T516) were substituted by alanine or phenylalanine (S727A, S521A, Y705F, amd T516A), which could not be phosphorylated or activated. As shown in Fig. 6H, compared with that of STAT3-WT, the phosphorylation of STAT3-S727A/S521A/ T516A was not affected, and the interaction between STAT3 and USP25 was not significantly changed. In contrast, the phosphorylation of STAT3-Y705F was significantly reduced, and the interaction between USP25 and STAT3 was significantly attenuated. Furthermore, after the mutation of tyrosine 705, USP25-catalyzed deubiquitination of K48-linked STAT3 was attenuated, and ubiquitination of STAT3 was increased, suggesting that USP25 catalyzed the K48-linked deubiquitination of p-STAT3^Y705^ (Fig. 6I).

### STAT3 alleviates ulcerative colitis in vitro and in vivo

**Fig. 7:**
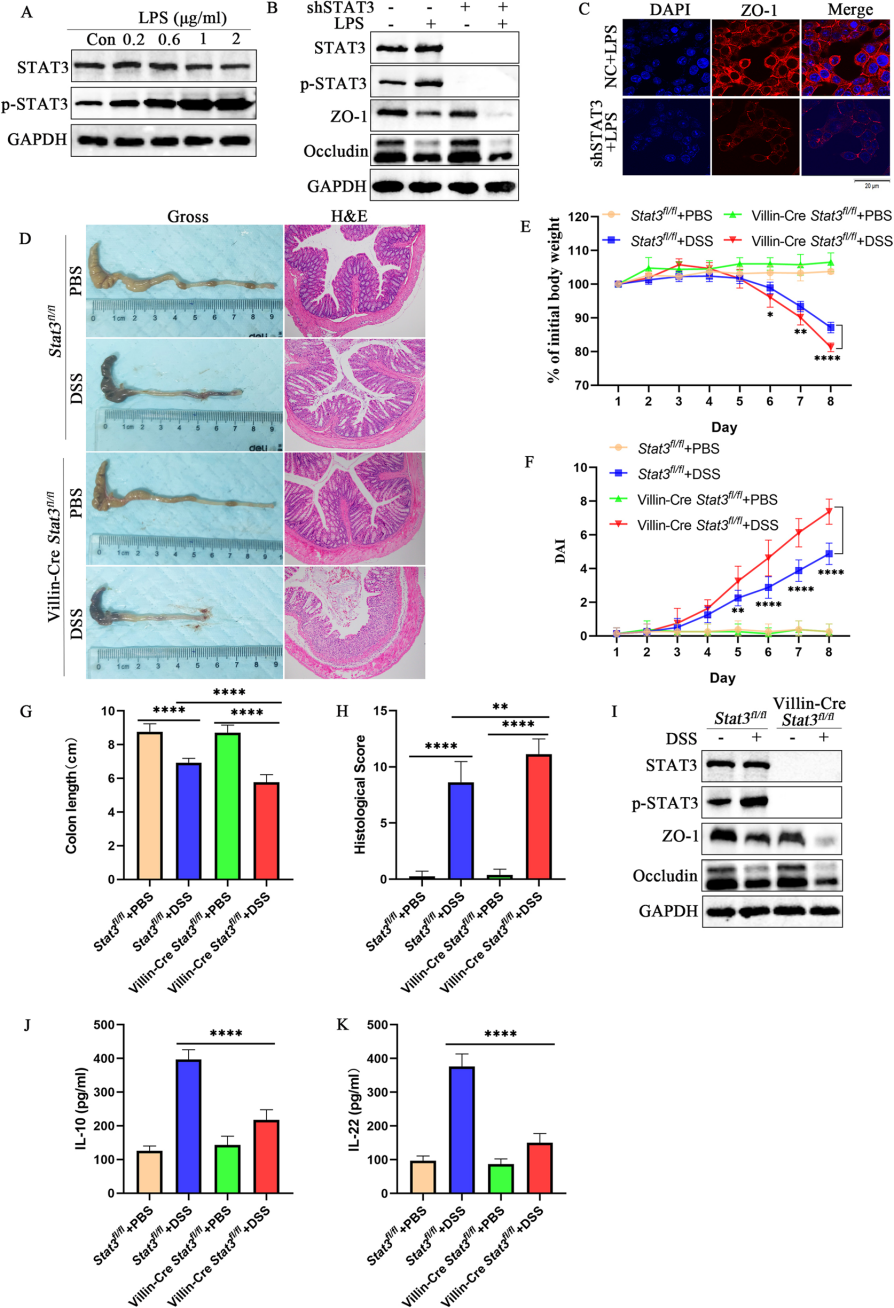
STAT3 alleviates ulcerative colitis in vitro and in vivo. **A** To determine the optimal LPS concentration, LPS was used to induce cellular inflammation in the human colonic epithelial cell line, NCM460. **B** After using lentivirus shSTAT3 to interfere with the expression of STAT3 in NCM460 cells, cells were co-cultured with LPS. Western blotting was performed to examine the protein expression levels of USP25, ZO-1 and occludin. **C** Immunofluorescence was performed to examine the expression of ZO-1 protein in NC and STAT3 low-expressing NCM460 cells with LPS treatment. **D** DSS was used to induce colitis in *State*^*fl/fl*^ and Villin-Cre *State*^*fl/fl*^ mice (*n* = 8 per group). Colons were photographed and recorded and stained with hematoxylin and eosin (*n* = 8 per group). Induction of colitis in mice was assessed by changes in mouse body weight (**E**), DAI score (**F**), colon length (**G**), and histopathological score (**H**). Body weight changes in mice are expressed as a percentage of the initial body weight (**E**). **I** Western blotting was performed to examine the protein expression levels of USP25, ZO-1 and occludin in colons from the four groups. **J**, **K** Serum IL-10 and IL-22 levels in *State*^*fl/fl*^ and Villin-Cre *State*^*fl/fl*^ mice (with or without DSS treatment) were measured by enzyme-linked immunosorbent assay. *p < 0.05, **p < 0.01, and ****p < 0.0001 compared with control groups.

To verify the role of STAT3 in UC, LPS was used to induce inflammation in NCM460. The results showed that when the concentration of LPS was 1 μg mL^−1^, STAT3 was significantly phosphorylated (*p* < 0.05) (Fig. 7A). In addition, after using lentivirus shSTAT3 to interfere with the expression of STAT3, NCM460 cells were co-cultured with LPS, and the expression of ZO-1 and occludin in cells with low STAT3 expression were significantly reduced (*p* < 0.05) (Fig. 7B, C).

Since *Stat3*-knockout mice may undergo embryonic death, we bred STAT3 intestinal epithelial cell-specific knockout mice (Villin-Cre *Stat3*^*fl/fl*^). Compared with *Stat3*^*fl/fl*^ mice, *Stat3*-deficient (Villin-Cre STAT3^fl/fl^) UC mice showed significantly shorter colon length (*p* < 0.05), significant weight loss (*p* < 0.05), and increased DAI (*p* < 0.05) and histopathological scores (*p* < 0.05) (Fig. 7D–H). In addition, *Stat3*-deficient UC-group mice also showed more severe tight junction damage than *Stat3*^fl/fl^ UC mice (*p* < 0.05) (Fig. 7I). Moreover, serum levels of anti-inflammatory factors IL-10 and IL-22 in *Stat3*-deficient UC mice were lower than those in the *Stat3*^*fl/fl*^ UC mice (Fig. 7J, K). Thus, STAT3 deficiency aggravates DSS-induced colitis in mice. In summary, STAT3 alleviates inflammation in UC.

### STAT3 overexpression by adeno-associated virus attenuates colitis in DSS-induced *Usp25*^*−/−*^ mice

**Fig. 8:**
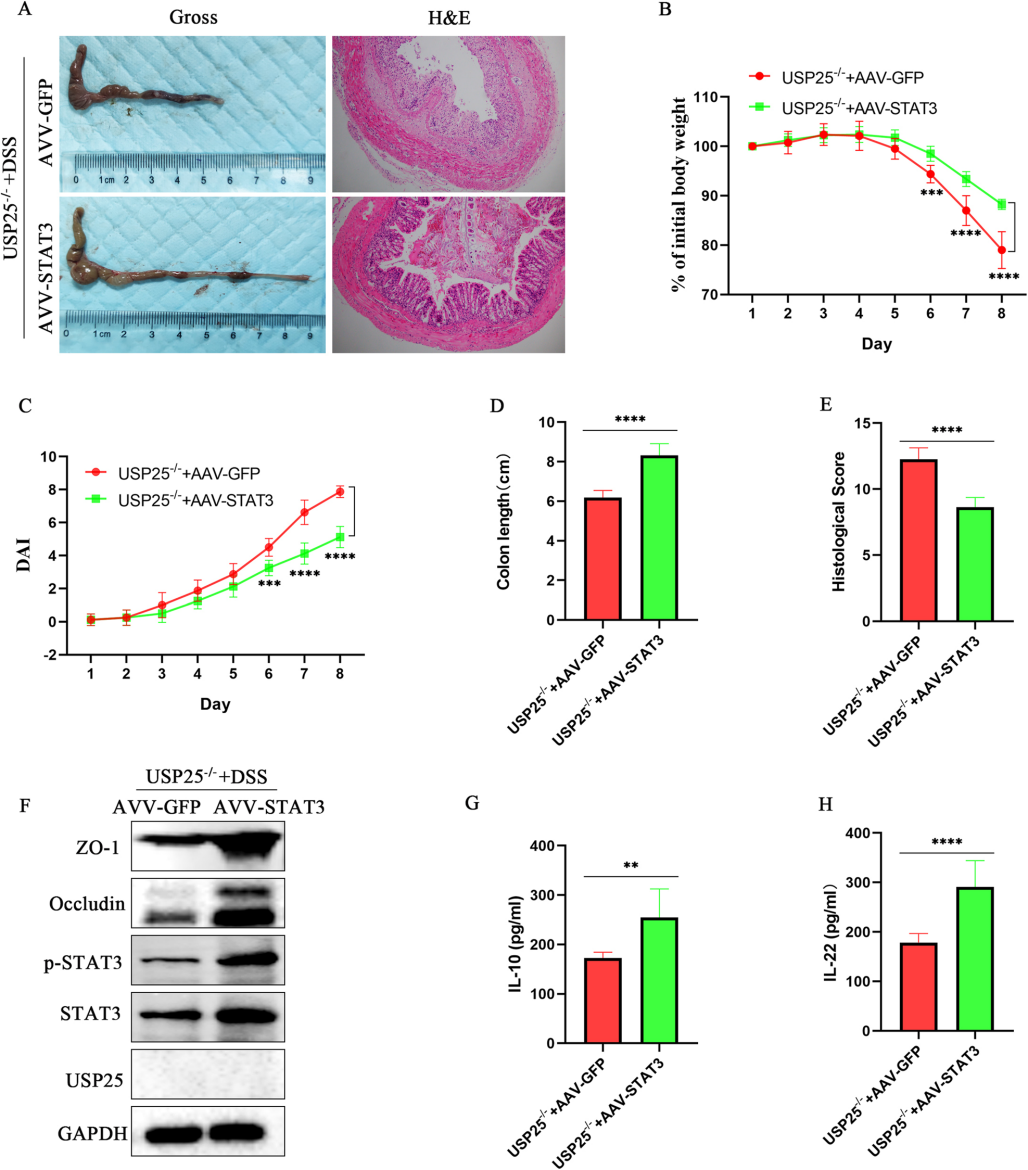
STAT3 overexpression by adeno-associated virus attenuates colitis in DSS-induced *Usp25*^*−/−*^ mice. **A** Adeno-associated virus 8 (AAV8) expressing STAT3 (AAV–*Stat3*) was injected into mice to overexpress STAT3 and subsequently induce colitis in *Usp25*^*−/−*^ mice with DSS. Colons were photographed and recorded and stained with hematoxylin and eosin (n = 8 per group). Induction of colitis in *Usp25*^*−/−*^ mice was assessed by changes in mouse body weight (**B**), DAI score (**C**), colon length (**D**), and histopathological score (**E**). Body weight change in mice was expressed as a percentage of the initial body weight (**B**). **F** Western blotting was performed to examine the protein expression levels of USP25, STAT3, p-STAT3, ZO-1 and occludin in colons from the mice in two groups. **G**, **H** The levels of mouse serum inflammatory factors (IF) IL-10 and IL-22 were measured by enzyme-linked immunosorbent assay. **p < 0.01, ***p < 0.001, and ****p < 0.0001 compared with control groups.

Finally, we examined the therapeutic efficacy of STAT3 in UC. We injected adeno-associated virus 8 (AAV8) expressing STAT3 (AAV-*Stat3*) into DSS-induced *Usp25*^*−/−*^ mice. Compared to the control group (AAV-GFP), the AAV*Stat3*-injected *Usp25*^*−/−*^mice exhibited longer colons (*p* < 0.05), less weight loss (*p* < 0.05), lower DAI scores (*p* < 0.05) and histopathological scores (*p* < 0.05), and less tight junction disruption (*p* < 0.05) (Fig. 8A–F). Moreover, serum levels of anti-inflammatory factors IL-10 and IL-22 in the AAV*Stat3*-injected *Usp25*^*−/−*^mice were higher than those in the control group (Fig. 8G, H).

## Discussion

More than 100 genes encode deubiquitinase (DUBs) in the human genome, and DUBs include the following six categories: USPs, ubiquitin carboxy-terminal hydrolases, ovarian tumor proteases, Josephine family, ubiquitin-interacting enzyme motifs, and zinc-dependent JAB1/MPN/MOV34 metalloprotease DUBs (JaMs) [25]. USP25 is a member of the USP family and plays an important role in the regulation of protein degradation, cell cycle, immunity, and DNA repair pathways [26]. A genome-wide association study, which identified African-specific susceptibility loci in African Americans with inflammatory bowel disease, showed that USP25 is closely related to the pathogenesis of IBD [5]. We downloaded three datasets from the GEO database, and the statistical results confirmed that the expression of USP25 in patients with UC was lower than that in healthy individuals. This result was further confirmed in human colonic tissue, experimental mice, and cells. In addition, we confirmed the role of USP25 in alleviating intestinal inflammation in UC by knocking out or overexpressing USP25 in experimental mice and cells.

Intestinal epithelial cells (IECs) of the gut represent a physical barrier that separates the microbiota from the mucosa and integrates afferent signals from gut symbiotic bacteria, pathogens, and dietary components. The permeability of the barrier, balance between differentiation and renewal of IECs, and production of antimicrobial peptides are important factors for the integrity of IECs [27]. Cytokines, especially IL-10 and IL-22, play important regulatory roles in these processes. They promote the proliferation and survival of IECs by inducing the activation of STAT3, and the activation of STAT3 further promotes the secretion of IL-10 and IL-22 [20, 21, 28]. Therefore, the IEC-specific deletion of STAT3 exacerbates DSS-induced intestinal epithelial damage and impairs proliferation [28]. In our study, we also found that the IEC-specific deletion of STAT3 (Villin-Cre STAT3^fl/fl^) reduced the secretion of anti-inflammatory cytokines IL-10 and IL-22 and augmented epithelial erosions and mucosal barrier disruption after DSS-induced colitis.

Tight junctions are impermeable junctions that are commonly found in various epithelial and endothelial cells, capillary bile ducts, and renal tubules [29]. With the help of specific proteins, the adjacent cells are tightly connected. The tight junctions of colonic epithelial cells are critical for the colonic mucosal barrier, and colonic mucosal barrier damage is an important mechanism in the pathogenesis of UC [30]. Many types of tight junction-related proteins exist, and ZO-1 and occludin are commonly used in UC. Intestinal inflammation can lead to the destruction of colonic mucosal tight junctions, and the expression of ZO-1 and occludin proteins decreases [31, 32]. We also confirmed that the expression of tight-junction proteins ZO-1 and occludin was significantly downregulated in UC in vitro and in vivo. Moreover, we confirmed that USP25 and STAT3 upregulate the expression of ZO-1 and occludin to protect the intestinal mucosal barrier.

Proteins can be poly-ubiquitinated by E3 ubiquitin ligases for degradation, and the activity of E3 ubiquitin ligases can be counteracted by DUBs. USP25 directly interacts with tankyrases to promote their depolarization and stabilization, and then, tankyrases rapidly modify axin to promote axin proteolysis and β-catenin stabilization, thereby regulating the Wnt signaling pathway [33]. USP25 specifically reverses Lys48-linked TRAF3 ubiquitination to stabilize the tumor necrosis factor receptor-associated factor 3 (TRAF3) protein, thereby regulating the toll-like receptor signaling pathway [34]. In our study, we verified that USP25 interacts with STAT3 in UC using IP–MS and co–IP and confirmed that USP25 predominantly promoted K48-linked deubiquitination of STAT3. Subsequently, we found that STAT3 was deubiquitinated by USP25 at lysine 409 and that USP25 catalyzed K48-linked deubiquitination of p-STAT3^Y705^.

In conclusion, this study reveals that USP25 facilitates deubiquitination of p-STAT3, which subsequently inhibits destroying the intestinal mucosal barrier. Our study suggest that USP25 reduces the degradation of phosphor-STAT3^Y705^ at lysine 409 by catalyzing K48-linked deubiquitination and USP25 is a promising therapeutic target for the treatment of UC in the clinic.

## Supplementary information

Supplementary legends

Supplementary Figure 1

Supplementary Table 1

Supplementary Material

## Data Availability

All data relevant to the study are included in the article or uploaded as supplementary information.
