# Correction: Ubiquitin-specific protease 25 ameliorates ulcerative colitis by regulating the degradation of phosphor-STAT3

**DOI:** 10.1038/s41419-025-07491-6

**Published:** 2025-03-26

**Authors:** Zhengru Liu, Jian Liu, Yuping Wei, Jinting Li, Jixiang Zhang, Rong Yu, Qian Yang, Yinglei Miao, Weiguo Dong

**Affiliations:** 1 Department of Gastroenterology, Renmin Hospital of Wuhan University, Wuhan, 430060 China; 2 Department of Gastroenterology, The First Affiliated Hospital of Kunming Medical University, Kunming, 650032 China; 3 Department of Gastroenterology, The Third People’s Hospital of Chengdu, Chengdu, 610000 China; 4 Department of Gastroenterology, Guizhou provincial people’s hospital, Guiyang, 550002 China

**Keywords:** Disease model, Ulcerative colitis

Correction to: *Cell Death and Disease* 10.1038/s41419-024-07315-z, published online 07 January 2025

After the article was published, we found that a picture in Figure 3 (3A-6 group OE-HE) in the article was misused. This has been corrected.

Corrected Fig. 3 (revised figure)
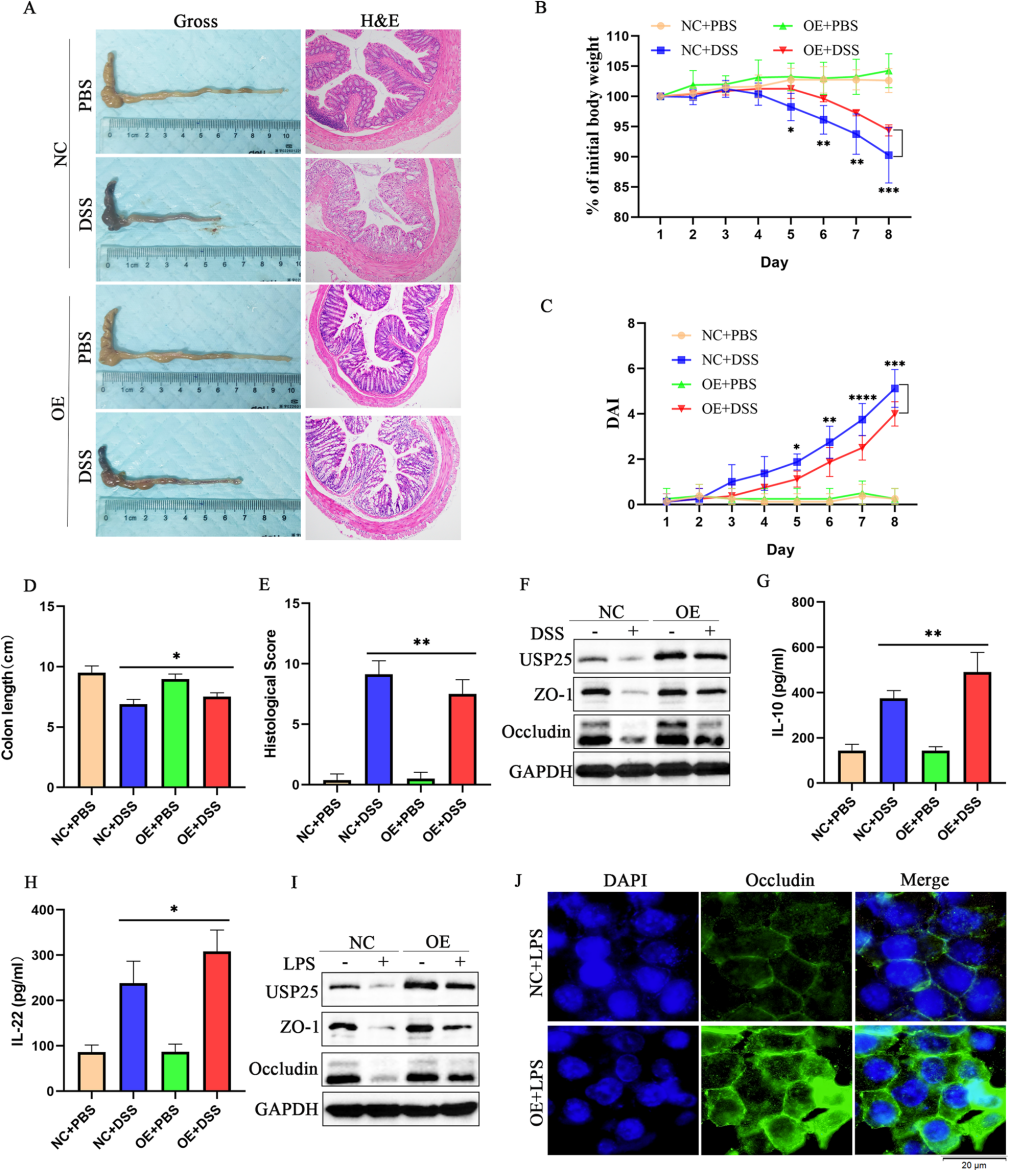


Incorrect Fig. 3 (originally published figure)
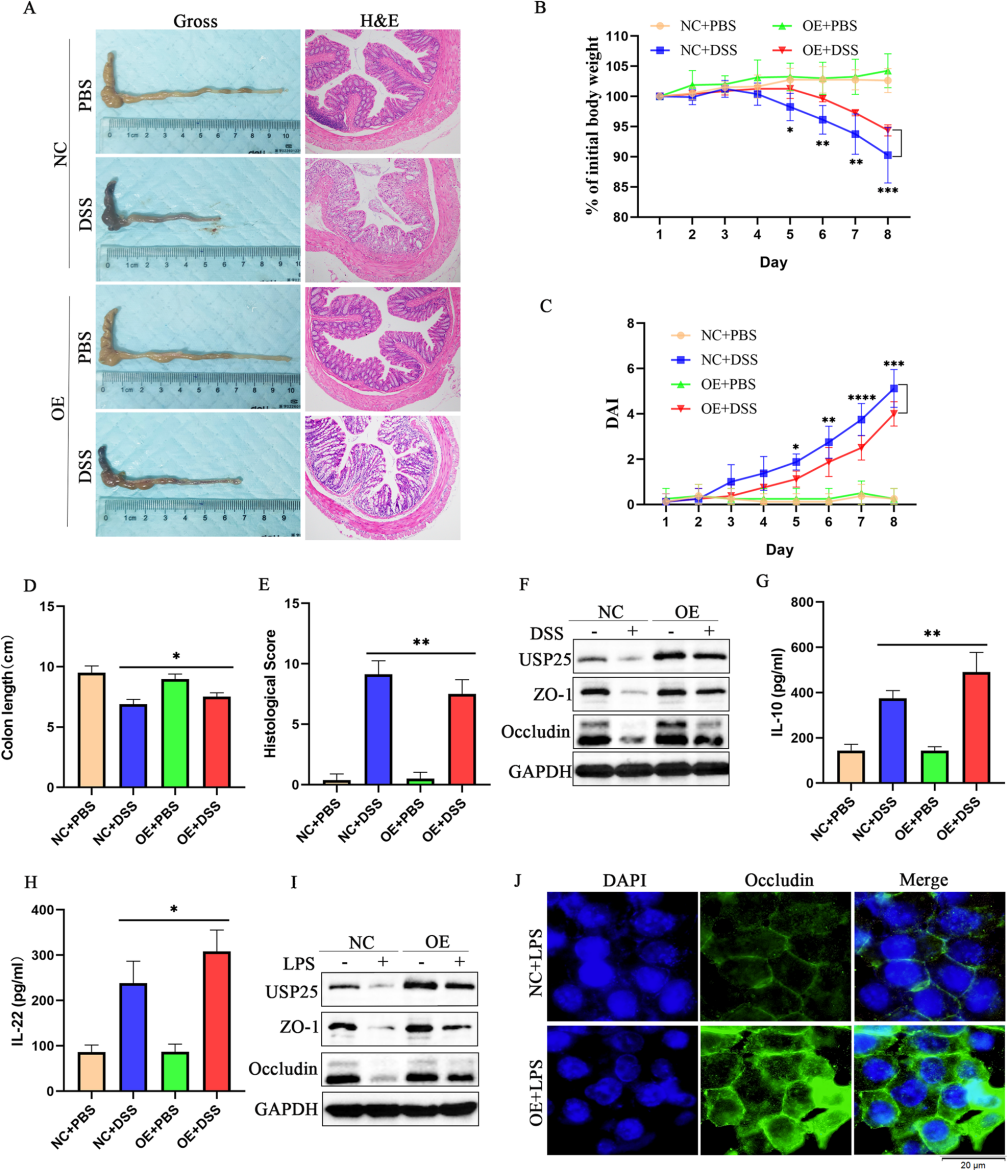


The original article has been corrected.

