# Supplementary material for: Ubiquitin-specific protease 25 ameliorates ulcerative colitis by regulating the degradation of phosphor-STAT3
Source: Cell Death Dis. 2025 Jan 7;16(1):5. doi: 10.1038/s41419-024-07315-z (PMC11707020; doi:10.1038/s41419-024-07315-z)
Supplement: Supplementary file 1 — Supplementary legends [file 41419_2024_7315_MOESM1_ESM.docx]

**Supplementary Figure 1. USP25 ameliorates ulcerative colitis by regulating the degradation of phosphor-STAT3**

A. Protein expression levels of USP25 in the colons of control and patients with UC, examined by immunohistochemistry, were quantified and compared.

B. Protein expression levels of ZO-1 in the colon tissues of control and colitis mice, examined by immunofluorescence, were quantified and compared.

C. Protein expression levels of USP25 in the colon tissues of control and colitis mice, examined by immunohistochemistry, were quantified and compared.

D. Protein expression levels of ZO-1 in control and USP25 low-expressing NCM460 cells, with or without LPS treatment, were quantified and compared.

E. To identify *Usp25^WT^* and *Usp25^-/-^* genotype of experimental mice, 2 % agarose gel electrophoresis was performed.

F, G. Levels of WT and *Usp25^-/-^* mice (with or without DSS treatment) serum IL-10 and IL-22 were measured using ELISA.

H. To identify *Stat^WT^* and Villin-Cre *State^fl/fl^* genotypes in experimental mice, 2 % agarose gel electrophoresis was performed.

I. Protein expression levels of p-STAT3 in LPS-treated NCM460 cells, with or without USP25-low-expression, examined by immunofluorescence, were quantified and compared.

**Supplementary Table 1. Substrate proteins of USP25**

Co-IP combined with MS was used to screen potential substrate proteins of USP25, the table shows all the substrate proteins of USP25 and corresponding scoring.
