# Supplementary figures and images for: Ubiquitin-specific protease 25 ameliorates ulcerative colitis by regulating the degradation of phosphor-STAT3
Source: Cell Death Dis. 2025 Jan 7;16(1):5. doi: 10.1038/s41419-024-07315-z (PMC11707020; doi:10.1038/s41419-024-07315-z)

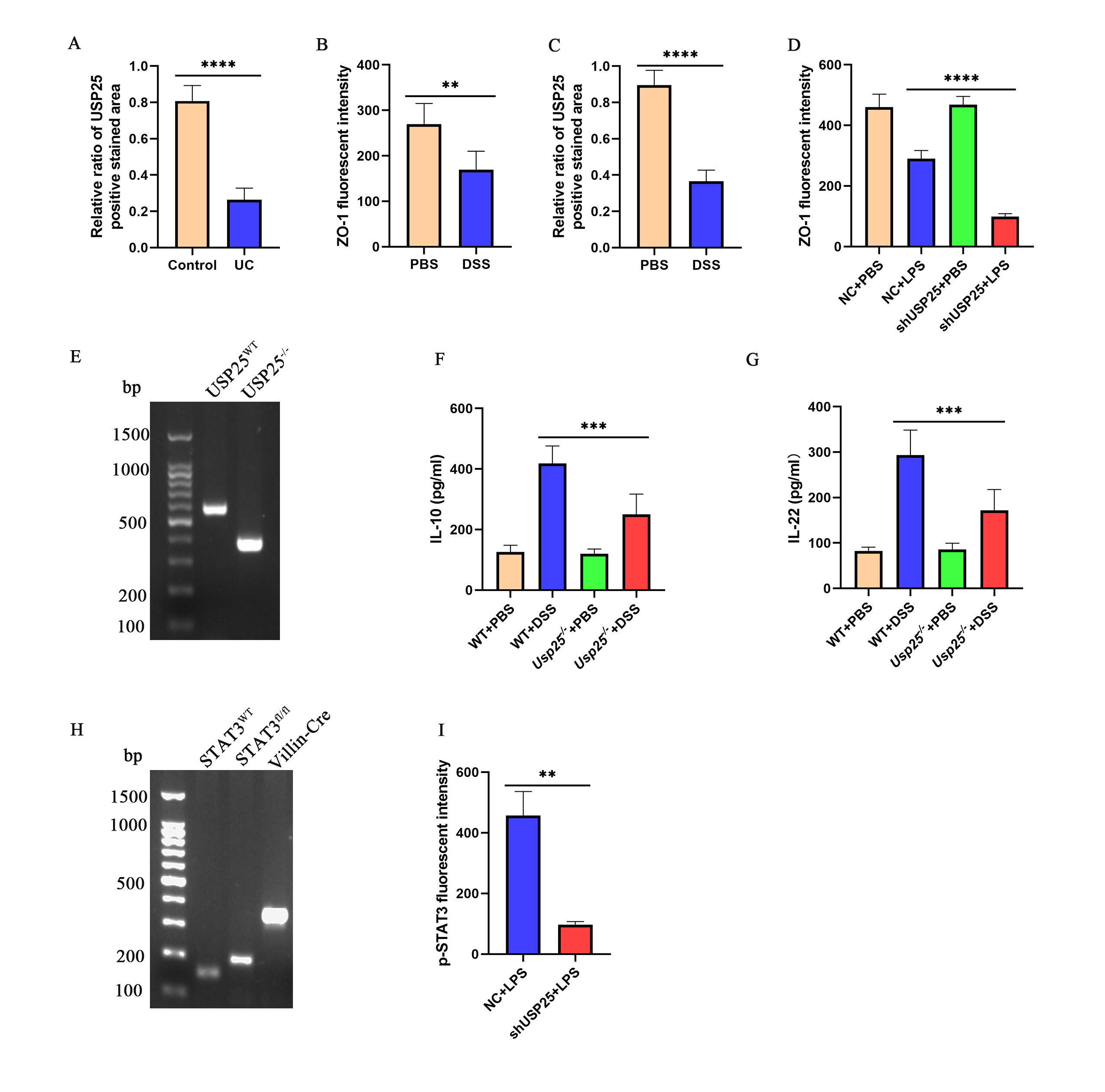

Supplement: Supplementary file 2 — Supplementary Figure 1 [file 41419_2024_7315_MOESM2_ESM.jpg]
