# Supplementary material for: Ubiquitin-specific protease 25 ameliorates ulcerative colitis by regulating the degradation of phosphor-STAT3
Source: Cell Death Dis. 2025 Jan 7;16(1):5. doi: 10.1038/s41419-024-07315-z (PMC11707020; doi:10.1038/s41419-024-07315-z)
Supplement: Supplementary file 4 — Supplementary Material [file 41419_2024_7315_MOESM4_ESM.docx]

Figure 1


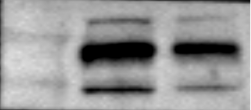
USP25


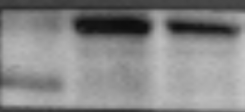
ZO-1


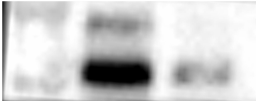
Occludin


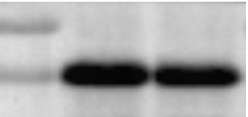
GAPDH

Figure 2

A


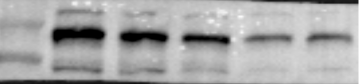
USP25


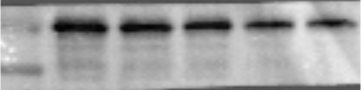
ZO-1


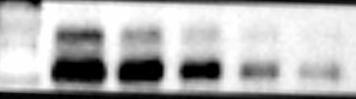
Occludin


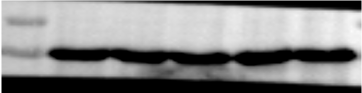
GAPDH

B


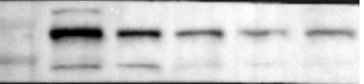
USP25


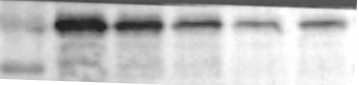
ZO-1


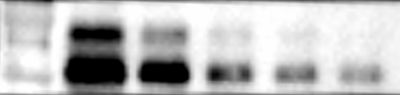
 Occludin


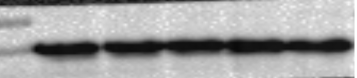
GAPDH

C


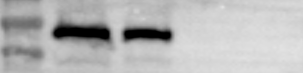
USP25


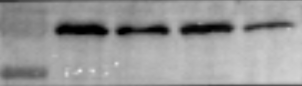
ZO-1


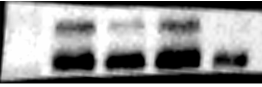
Occludin


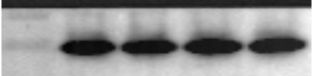
GAPDH

J


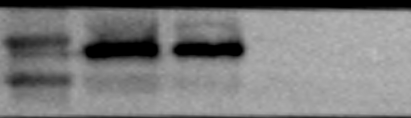
USP25


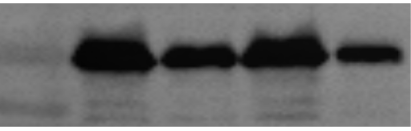
ZO-1


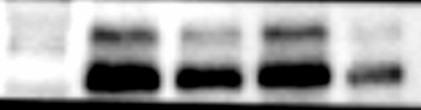
Occludin


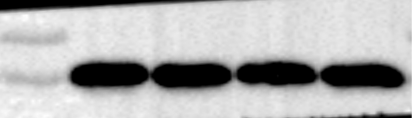
GAPDH

Figure 3

F


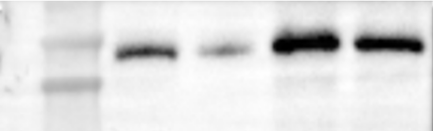
USP25


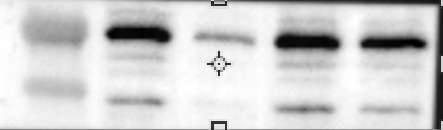
ZO-1


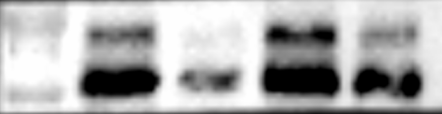
Occludin


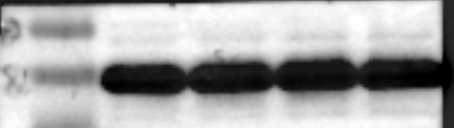
GAPDH

I


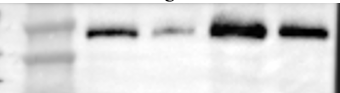
USP25


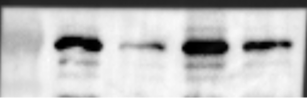
ZO-1


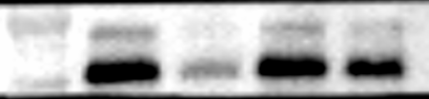
Occludin


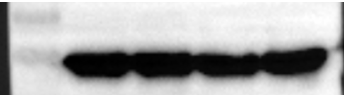
GAPDH

Figure4

A


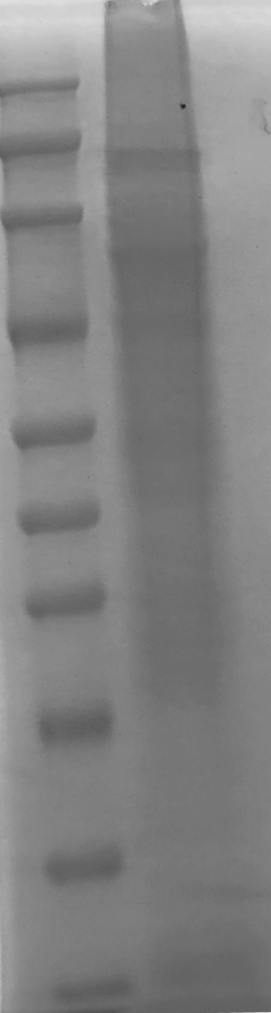
IP-Flag

B


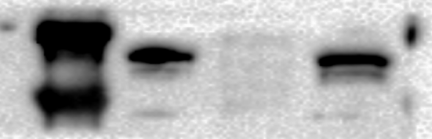
HA


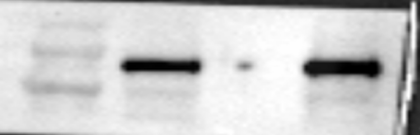
Flag

C


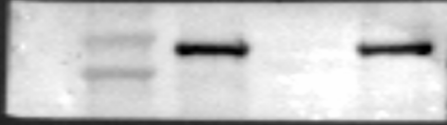
Flag


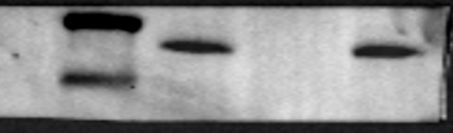
HA

D


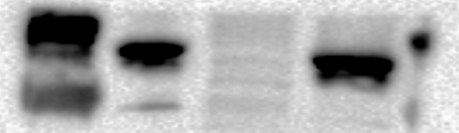
STAT3


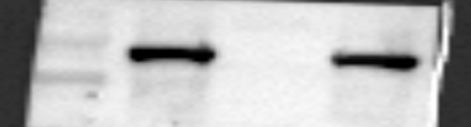
USP25

E


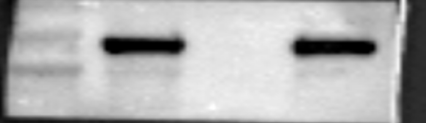
USP25


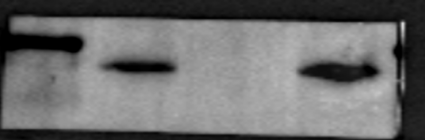
STAT3

Figure 5

A


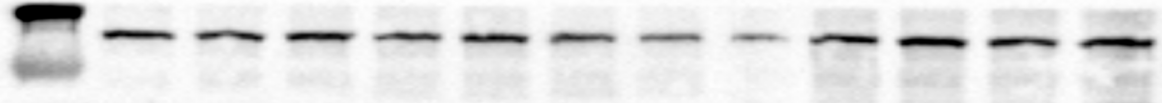
HA


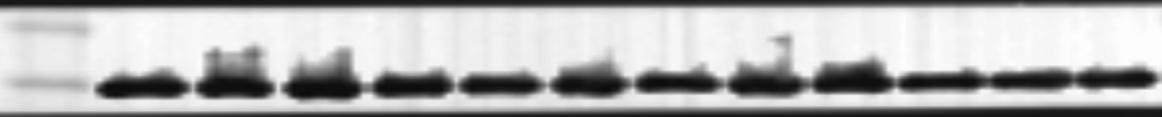
GAPDH

B


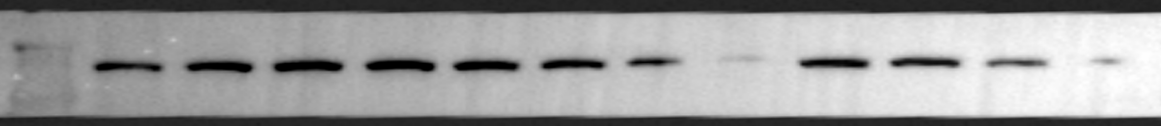
HA


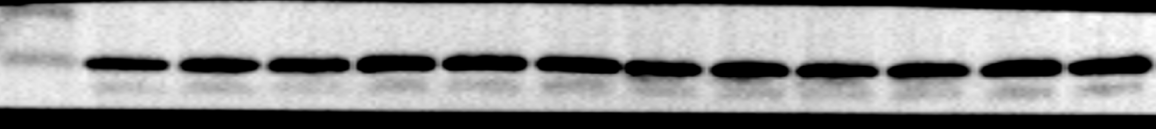
GAPDH

C


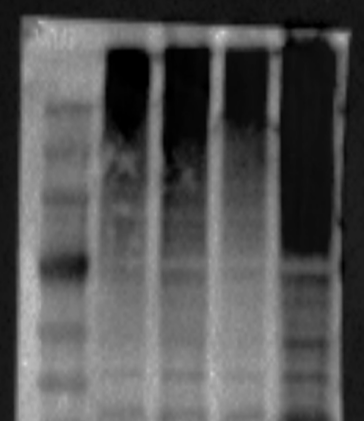
Ub


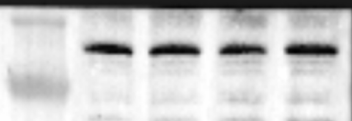
STAT3


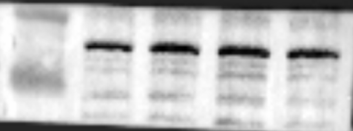
STAT3


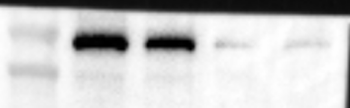
USP25

D


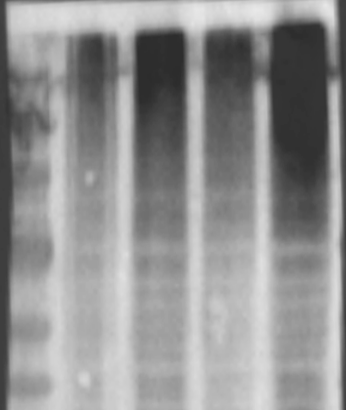
Ub


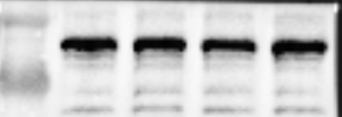
STAT3


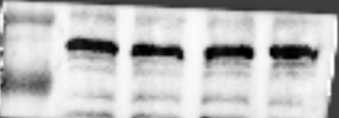
STAT3


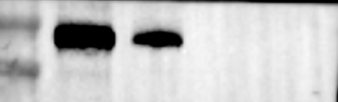
USP25

E


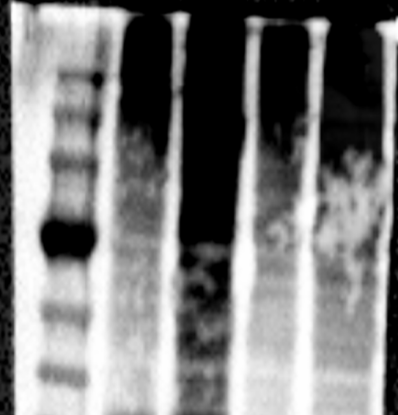
Ub


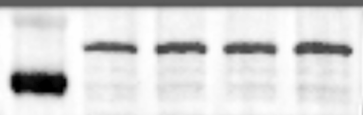
STAT3


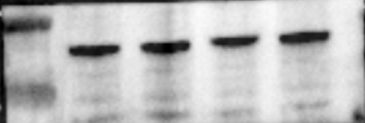
STAT3


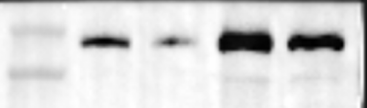
USP25

F


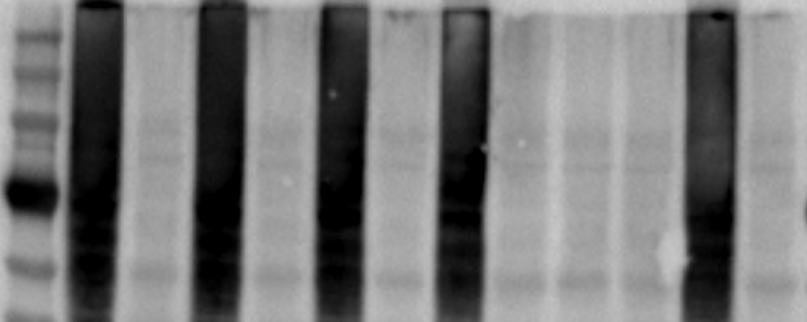
Myc


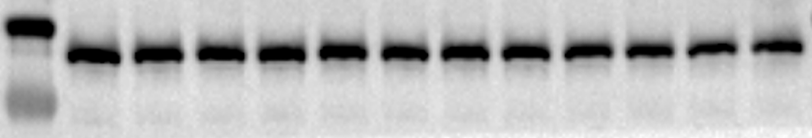
HA


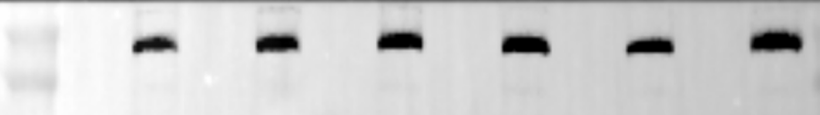
Flag


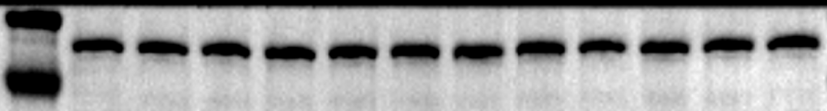
HA

G


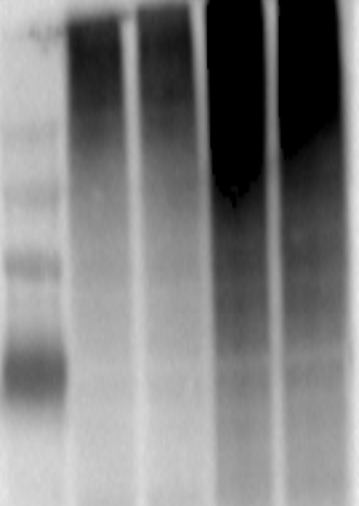
K48-Ub


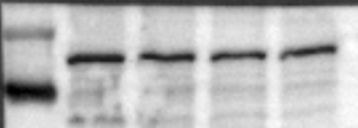
STAT3


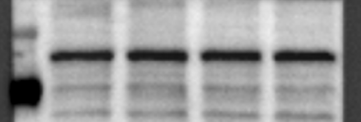
STAT3


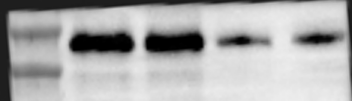
USP25

H


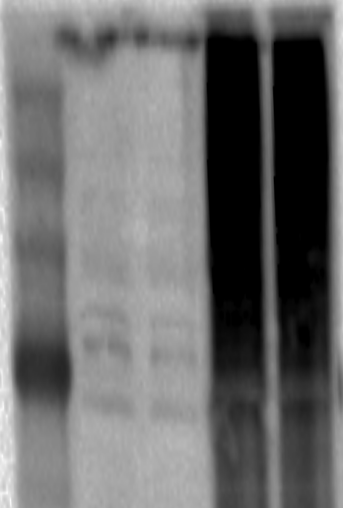
K48-Ub


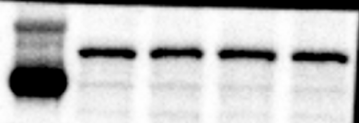
STAT3


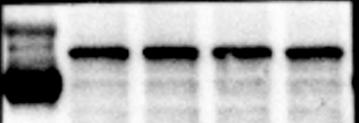
STAT3


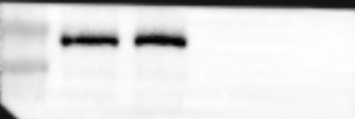
USP25

I


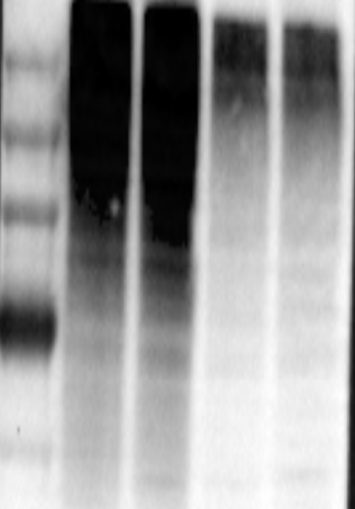
K48-Ub


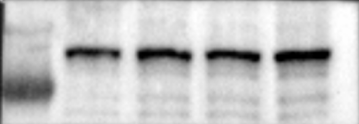
STAT3


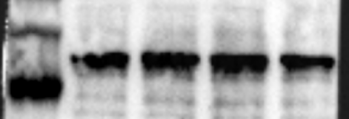
STAT3


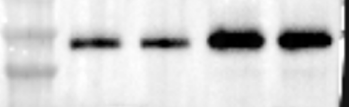
USP25

J


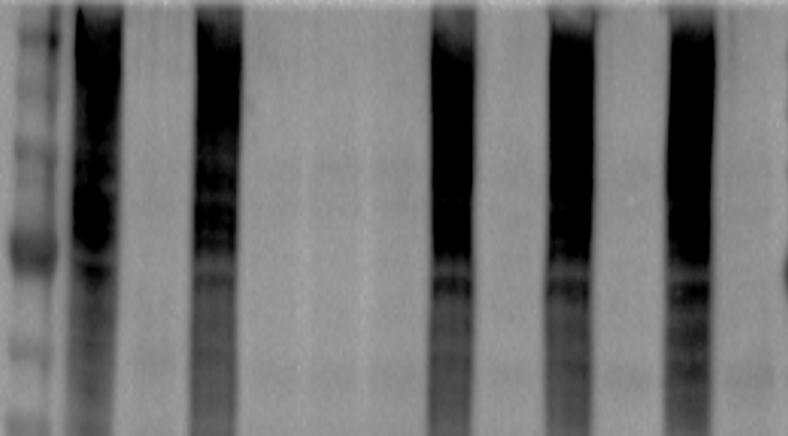
Myc


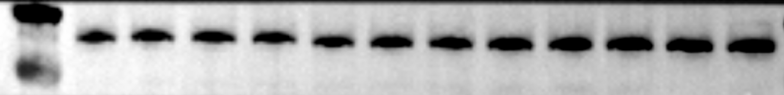
HA


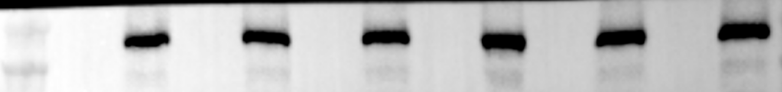
Flag


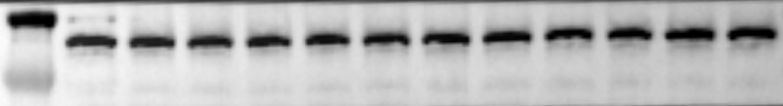
HA

Figure 6

B


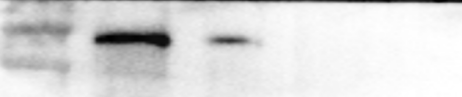
USP25


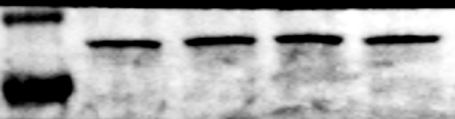
STAT3


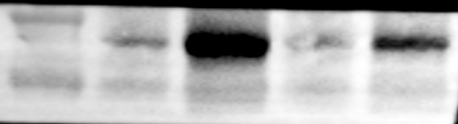
p-STAT3


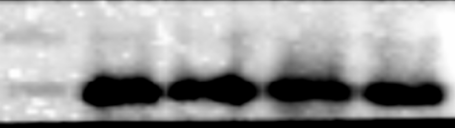
GAPDH

C


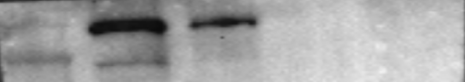
USP25


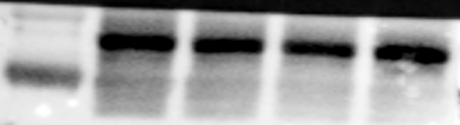
STAT3


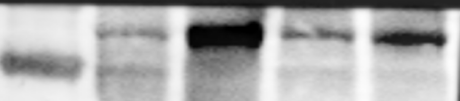
p-STAT3


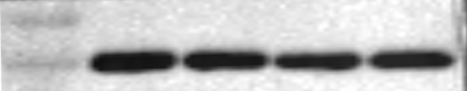
GAPDH

D


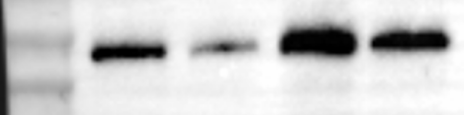
USP25


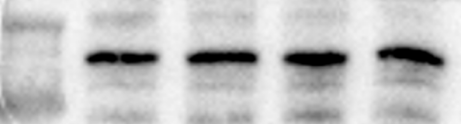
STAT3


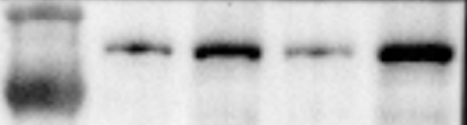
p-STAT3


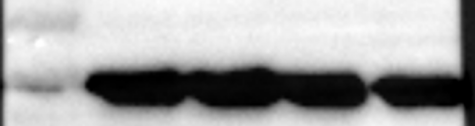
GAPDH

E


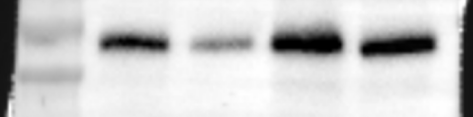
USP25


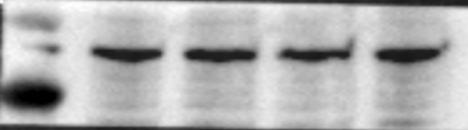
STAT3


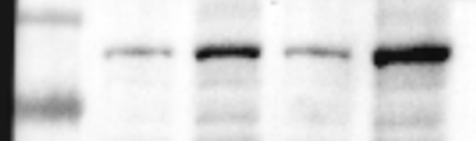
p-STAT3


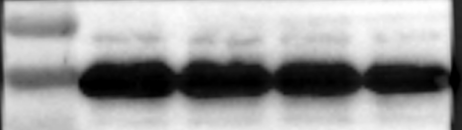
GAPDH

F


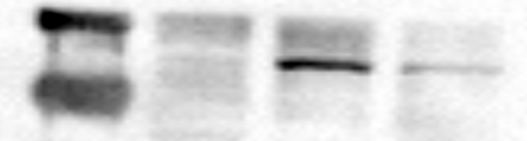
HA


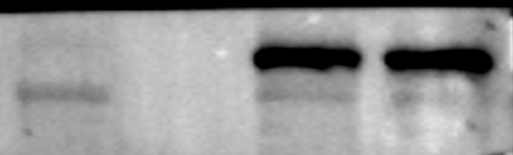
Flag


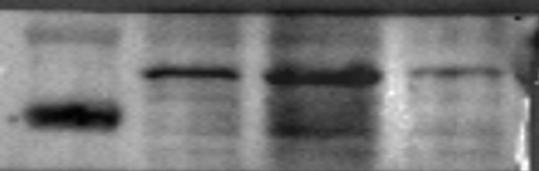
p-STAT3


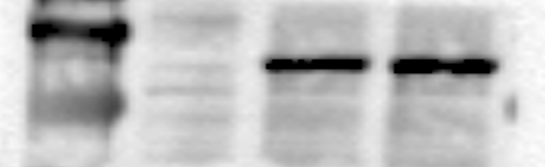
HA


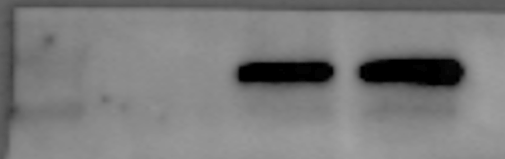
Flag


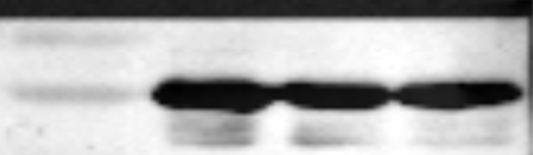
GAPDH

G


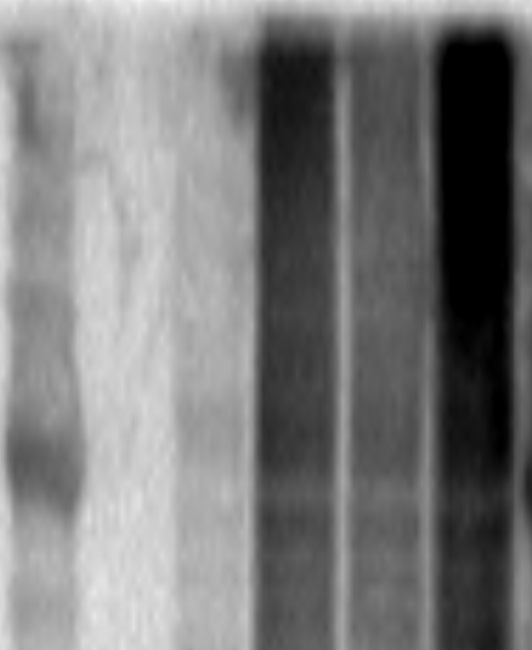
Myc


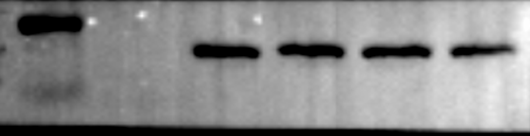
HA


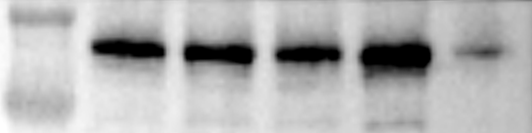
p-STAT3


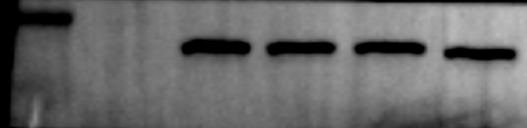
HA


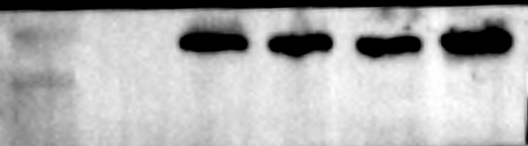
Flag

GAPDH

H

HA

Flag

p-STAT3

HA

Flag

GAPDH

I

Myc

HA

Flag

HA

Figure 7

A

STAT3

p-STAT3

GAPDH

B

STAT3

p-STAT3

ZO-1

Occludin

GAPDH

I

STAT3

p-STAT3

ZO-1

Occludin

GAPDH

Figure8

ZO-1

Occludin

p-STAT3

STAT3

USP25

GAPDH
